# Adaptive fault tolerance mechanisms for ensuring high availability of digital twins in distributed edge computing systems

**DOI:** 10.1038/s41598-025-25590-4

**Published:** 2025-11-24

**Authors:** Dinesh Sahu, Nidhi, Shiv Prakash, Tiansheng Yang, Rajkumar Singh Rathore, Lu Wang, Usha Sharma, Idrees Alsolbi

**Affiliations:** 1 SCSET, Bennett University, Plot Nos 8, 11, TechZone 2, Greater Noida, Uttar Pradesh 201310 India; 2 Department of Electronics and Communication, University of Allahabad, Prayag Raj, Uttar Pradesh India; 3 University of South Wales, Pontypridd, UK; 4 Cardiff School of Technologies, Cardiff Metropolitan University, Cardiff, UK; 5 Xi’an Jiaotong-Liverpool University, Suzhou, China; 6 Department of Information Technology, Babu Banarasi Das Institute of Technology and Management (BBDITM), Lucknow, India; 7 Data Science Department, College of Computing, Umm Al-Qura University, 21955 Makkah, Saudi Arabia

**Keywords:** Adaptive fault tolerance, Digital twins, Distributed edge computing, High availability, Hybrid genetic-PSO algorithm, Resource reallocation, Task migration, Node failure recovery, System resilience, Energy-efficient computing, Computer science, Information technology

## Abstract

The increasing adoption of Digital Twins (DTs) in distributed edge computing systems necessitates robust fault tolerance mechanisms to ensure high availability and reliability. This paper presents an adaptive fault tolerance framework designed to maintain the continuous operation of DTs in dynamic and resource-constrained edge environments. The primary objective is to mitigate failures at edge nodes, minimize downtime, and ensure seamless migration of DT instances without disrupting system performance. The proposed framework integrates a novel Hybrid Genetic-PSO for Adaptive Fault Tolerance (HGPAFT) algorithm, combining the strengths of genetic algorithms and particle swarm optimization. The algorithm dynamically reallocates resources and migrates DT instances in response to node failures, utilizing real-time monitoring and predictive failure detection to enhance system resilience. A key innovation lies in the adaptive nature of the fault tolerance mechanisms, which adjust resource reallocation and task migration strategies based on the evolving conditions of the edge network, such as node load, energy constraints, and communication delays. The results, validated through extensive simulations, demonstrate significant improvements in system availability, with recovery probabilities exceeding 98% and up to 20% reductions in reallocation and migration costs compared to traditional fault tolerance mechanisms. Additionally, the proposed framework optimizes energy consumption and resource utilization, critical for sustainable edge computing. This research contributes to the state of the art by offering a scalable and energy-efficient fault tolerance solution tailored for the decentralized and heterogeneous nature of distributed edge computing, ensuring the continuous and reliable operation of Digital Twins.

## Introduction

The concept of edge computing is new and has impacted the way data is managed, communicated, processed, and analyzed, particularly in use cases where instantaneous decisions and low latency are crucial^1^. One of the technologies that have taken advantage of edge computing includes Digital Twins (DTs), these are virtual models that represent the real system, and provide an update of the current state of actual objects in real time^2^. From things like entire city populations and the advanced fields of medicine and health, through production fields and linkages of intelligent autonomous cars, DTs give performance predictability, optimization, and better decisions^3^. Nevertheless, the performance gains of DTs substantially depend on the constant accessibility and dependability of the DT across distributed edge computing scenarios^4^. Given that edge networks are decentralized, dynamic and resource constrained it is challenging to maintain high availability of DTs, which leads to the consideration of fault tolerance as a major problem^5,6^.

**Fig. 1:**
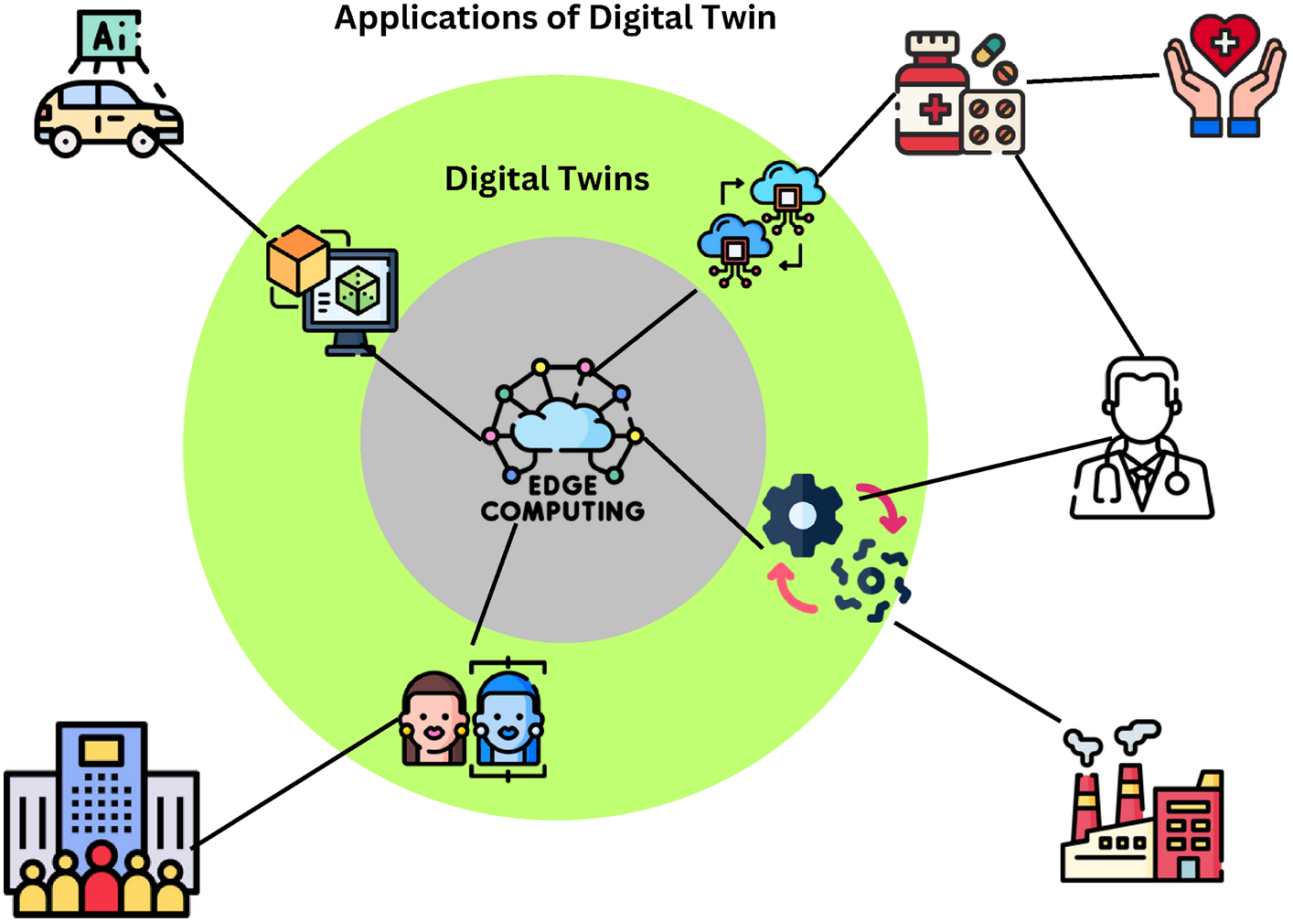
Digital twin assisted edge computing.

The Fig. 1 highlights a structural pattern of Digital Twins (DTs), as well as their uses and issues related to edge computing. Fundamentally, Edge Computing remains the underlying technology on whichDT relies on for processing data in real time and with low latencies. The last node, Digital Twins, expands into various application areas – City Populations, Medicine and Healthcare, Production Fields, and Intelligent Autonomous Car to illustrate how a concept can be ubiquitous normalizing issues of system performance and predictability. Also, the graph depicts issues like Accessibility, Dependability, and Fault Tolerance labelled alongside DTs as the areas of focus that are required to ensure the sustainment of high availability and reliable solutions in distributed and resource-scarce edges. The structure visually shows the huge opportunity if one decides to venture in DTs but at the same time show the need to have very strong backup mechanisms because of possible operations constrains. Digital Twins (DTs) in distributed edge computing settings are associated with new challenges because the edge nodes are dynamic and resource-constrained and may also fail. DTs depend directly on physical assets and their ongoing synchronization and the integrity of data transfer, processing, or communication may affect the effectiveness of the DTs in terms of fidelity and real-time capability. In comparison with centralized cloud systems, edge environments are also more vulnerable to the hardware failures, network partitioning, interruption of energy sources, and over-utilization, that may result in the significant decrease of DT performance and availability. Accordingly, the final fault tolerance does not represent the secondary aspect of a functioning system since its efficient operation is an essential stipulation to the achievements of DT-based applications in smart production, networked health, autonomous vehicles, and industrial IoT. It is clear that effective fault-tolerant mechanisms have to be adaptive, lightweight, and energy-efficient to seamlessly operate DTs in conditions of a varying system load without producing excessive overhead.

### Problem statement

Due to the dispersed nature of computational resources in distributed edge computing systems, easy availability of the digital twin remains critical to achieving tight recovery time objectives between the physical assets and their virtual counterparts^7,8^. Any breakout of this synchronization that emanates from node failures, network breach, among other mishaps could lead to poor performances, wrong predictions, and in other instances disastrous incidents in real-time sensitive application^8^. Since edge computing nodes are generally implemented in resource constrained environments (for instance in IoT or remote industrial ecosystems), they are more sensitive to failure than centralized computing nodes because of low density resources, energy limitations, and connectivity instabilities^9,10^. The requirement for building a functional fault tolerance mechanism that is capable of identifying failure, handling it, and recovering from it all while ensuring the operation of DTs, is important^11^.

### Motivation

Reliability therefore is a critical component that should be incorporated in the designing and implementation of edge computing systems due to its susceptibility to possible failure^12^. In distributed environments these failures may result from a subtle cause, for instance hardware failure, energy drain, or communication breakdown between nodes. In the absence of strong fault tolerance, the failure of a single edge node may have a system crashing effect, which means that for a digital twin system which is hosted in an edge node, its failure implies loss of data integrity, lost opportunities of doing real time analysis, as well as the knock on effect on other systems depending on it^13,14^.

The rather complex structure of the edge infrastructure is due to the fact that the nodes have diverse computational and memory capacity and power consumption rates, and it only adds to the challenges of fault management^15,16^. Unlike the cloud data centers where resources are effectively centralized flexibly and in abundance, the edge nodes are in effect decentralized having dynamic workload and variable resources^16^. Thus, fault tolerance strategies have to be able to compensate for faults quickly and not spend too much time or resources doing so. Furthermore, DTs must be updated in real-time and in real-time they depend on available edge nodes, and even brief outages of these end nodes can lead to severe influences on the prediction accuracy of the DT and functionalities^17^.

### Research gap

Current fault tolerance approaches in edge computing systems have several limitations that hinder their effectiveness when applied to digital twin environments^18,19^. Some of the classical fault tolerance measures are generic, including checkpoints, and replication of tasks where a set of rules is used in the unlikely event of a failure occurring. Although such approaches offer a simplistic form of fault recovery, they are most ineffective for the complex and distributed edge setting. Incorporation of static strategies does not factor in real time changes such as node availability, workload distribution or even resource conditions hence making recoveries and resource allocation sub-optimal^19,20^.

In addition, many of the current methods do not consider specific requirements of digital twins which are low latency, high availability, and synchronizing with the applied physical systems in real-time^20,21^. These approaches mainly focus on liability identification and restoration that are not necessarily optimized power consumption and usage of resources in the edge nodes which are indeed constrained with resources. An area still open to research is the development of adaptive fault tolerance solutions aware of system conditions which they then use for adjusting their operational procedures dynamically^22^.

### Contribution

In response to the challenges described above, this paper proposes a new framework for the adaptive fault tolerance mechanisms aimed at high availability of the digital twins in the distributed edge computing environment. The core contributions of this research include:

1. *Hybrid Genetic-PSO for Adaptive Fault Tolerance (HGPAFT) Algorithm* In this paper we present an adaptive fault tolerance approach that integrates the advantages of GA and PSO to come up with a new hybrid algorithm. Resource allocation and migration of digital twin instances take place at run time, thus mitigating the effects of node failure.
2. *Real-Time Fault Detection and Recovery* The system designed in our framework includes the use of preventive fault forecasting methods to reduce the incidence of failure. Due to setting up constant checks on the node’s health and load distribution, the system makes adjustments to the resource distribution.
3. *Energy-Efficient Fault Tolerance* The highly beneficial proposed approach also considers energy localization, how to allocate and reallocate resources and possibly migrating in order to avoid a recurrence of conking out incidences and at the same time avoiding damaging the fault tolerance mechanisms by consuming the energy of the nodes at the edges of the system.
4. *Comprehensive Performance Evaluation* Through extensive simulation, we assert the efficiency of the proposed framework in terms of recovery probability, cost of reallocation and migration, energy and resource utilization compared to traditional fault tolerance techniques.

### Paper structure

The organization of the rest of this paper is as follows. “Background and related work” section provides some background to this work in the form of a literature review, detailing the fault tolerance mechanisms in edge computing and digital twin. “System architecture and problem definition” section introduces the new adaptive fault tolerance framework in detail, and introduces the HGPAFT algorithm together with its incorporation of the real time fault identification process. “HGPAFT: hybrid genetic-PSO for adaptive fault tolerance” section presents the performance study of the proposed framework while “Evaluation and experimental setup” section compares and contrasts the outcome of the proposed framework with existing solution methodologies using simulations. Lastly, “Evaluation and experimental setup” section presents the implication of the study and possible use of the framework in different fields. Last but not least, “Results and discussion” section brings conclusion for this paper together with recommending directions for further research in adaptive fault tolerance for the edge computing systems.

To enhance the reliability and scale of digital twins in the context of distributed edge computing, this study proposes a novel method of fault tolerance that is adaptive to the demands.

## Background and related work

Digital twin technology that was proposed as a means to create a virtual replica of an existing product or object is being increasingly adopted more and more across industry. Combining them with edge computing makes it possible to perform data processing at the same time as decision-making near the data source with the help of digital twins to support such applications as predictive maintenance, intelligent manufacturing, or IoT solutions. Using edge computing, digital twins reside as a valuable and flexible tool to represent, analyze and manage physical assets via virtual model. While being beneficial for optimising operation, these digital twins also enable the ongoing supervision and improvement of devices and processes in real-world conditions. Edge computing offers this by offering the computational platform to do this by minimizing latency, increasing rates of response, and decentralizing processing near the physical asset^23,24^.

The use of digital twins with edge computing provides following benefits. It allows for local data processing, limited usage of centralized clouds, enhances system capacity, availability, and throughput^25^. However, achieving high availability and reliability of the associated digital twins in such a distributed and resource-limited setting raises new questions. Another issue is partial failures, that is whether the system continues to operate in the correct manner when there are faults in differing partitions^26^.

### Fault tolerance in distributed system

Fault tolerance mechanisms work to make systems allow repairs on possibly faulty hardware or software parts without compromise on services^27^. Nowadays, most real-world applications deploy distributed edge computing since it involves several nodes, and at times, these nodes may be exhausted of resources, get disconnected from a network, or encounter system-related bugs^28^. Other forms of fault tolerance widely used are replica, checkpoint and rollback recovery techniques. For instance, replication generates different duplicates of a single process or data at different nodes, and if one of such nodes has a glitch, another node will be prepared to take over the task . Checkpointing is a method where the system records a status of a process at regular intervals to enable an attempt at recovery if the process fails. Accordingly, rollback recovery brings the system back to a previously correct state whenever an error is identified^28,29^.

These are traditional methods which perform reasonably well in centralized systems or on the cloud but they become scaled up and require many resources which makes them inconsequential in low resource environments like the edge computing environment^30^. However, as the workload and network topology in edge environments can be more variable and volatile [8], then the fault tolerance strategies needed are more adaptive and lightweight^31,32^.

### Related work

Recent advancements in edge computing and digital twins have driven increased attention towards fault tolerance mechanisms, ensuring the reliability and availability of these systems^33^. discussed dynamic resource estimation and pricing models for IoT in fog computing, underlining the importance of resource management for fault tolerance.Digital Twins (DTs) combined with edge computing have now immensely transformed real-time monitoring and control in different areas. DTs reproduces physical object to provide real-time information analysis. DTs integration into edge computing environment works effectively improving the performance of the overall systems, as response time and latency are critical for smart cities, healthcare and industrial applications^34,35^. For example, a conceptual framework for the 6G Edge of Things (EoT) system includes integrating DTs to support the execution of low-latency services and real-time decision making^36–38^.

However, it is not easy to maintain a high availability of DTs in distributed edge computing situations. Since edge networks are inherently distributed and low on resources, eradicating faults and failures in such networks becomes a complex proposition^39^. In response to these problems, the researchers have had to delve into several fault-tolerant techniques. For instance, the DRAGON framework coordinates decentralized fault tolerance in edge federations using generative optimization networks to forecast and enhance performance^40^. Likewise, the DeepFT model applies a self-supervised deep surrogate model to establish fault tolerance in edge computing^41^. Self-adaptive mechanisms can be identified as one of the key enablers for improving fault tolerance in a DS. GAs and PSO are two major forms of techniques used in this regard due to their optimization feature. For example GAs mimic the natural selection to arrive at optimization solutions and PSO is based on the flocking behaviour of birds or shoaling of fishes^42^. There are also suggestions to integrate these algorithms to recognize the advantages for each of them. For instance, the use of the combined GA-PSO algorithm has been used in adaptive protection in power systems with added benefits of increased convergence speed and the capability of search than the two in isolation^43^. A similar study proposed a GA-PSO approach to fault-tolerant formation control of wheeled mobile robots and this effectively treated problems of convergence and search^44^. Further related works discuss about the IoT-Edge structures of consistent and failure-resistant for various purposes^45,46^.

Nevertheless, the above solutions are not without shortcomings in the current world. Most of the present fault tolerant approaches are infrastructured more for particular deployments and therefore may not be convenient in different edge computing paradigm. Also, the edge networks are continually evolving and hence call for dynamic fault tolerance mechanisms that can operate under different conditions^47,48^. Indeed, conventional algorithms may suffer from the problem of scalability and hence may not be well suited to effectively process large volumes of data produced in real-time by DTs. In addition, with the incorporation of DTs with edge computing, there are new challenges of updating and synchronizing the data for real-time representation^49^. These gaps need to be filled with the practical development of more general, large-scale, and robust fault tolerance mechanisms so that DTs can realize high availability and reliability in distributed edge computing system^50–52^.

Over the past years, various works have been carried out on the foundational aspects of serverless and edge computing that would be of importance to fault tolerance, scale and resource management^53^. carried out detailed review of proactive content caching strategies in edge settings and emphasized the significance of lowering service latency and preserving data availability both of which are important to enable fault-tolerant operation of DT. Other surveys that have been contributed by^54^ involve several important dimensions of serverless execution: approaches to placement functions^55^, scheduling^56^, and offloading functions^54^. The following works focus on necessity of dynamic, lightweight, and context-aware models of computing, which is consistent with the aim of adaptive fault tolerance of edge-based DT systems^57^. developed a methodical approach to offload energy-conservation computation in fog scenario which is identical to our aim to reduce resource overloading and response time in a failed scenario by a method similar to a hidden Markov model. Finally^58^, provided an extensive survey of auto-scaling in serverless systems, supporting the importance of scalable and elastic techniques, which are also found in the multi-objective optimization architecture of the framework developed, called HGPAFT. All these works diversify the base of our research direction and offer supplementary insights into the suggested methodology.

**Table 1:** Summary of related work on fault tolerance and resource management in edge-enabled DT systems.

Ref.	Tools/dataset	Metrics	Advantages	Limitations
^53^	Survey (edge caching)	Qualitative taxonomy	Structured view on caching strategies	No DT focus; lacks implementation
^54^	Function placement review	Placement delay, latency	Comprehensive function placement taxonomy	No fault tolerance or DT context
^55^	Serverless scheduling survey	Invocation delay, overhead	Insight into scheduling in dynamic loads	No DT consideration or failure handling
^56^	Serverless offloading review	Latency, execution time	Classifies fog/edge offloading methods	No DT or fault scenarios addressed
^57^	FogSim, HMM models	Latency, energy	Probabilistic offloading using HMM	Limited scalability; no hybrid models
^58^	Survey on scaling tools	Throughput, response time	Covers scaling strategies in edge clouds	No failure resilience or DT integration
HGPAFT	Python sim, 4 failure cases	Task success, energy, recovery time	DT-aware fault handling via GA-PSO; Pareto-optimal selection	Moderate CPU load; requires real-time monitoring

Table 1 presents a comparative analysis of the main related studies that deal with edge computing and Digital Twin (DT) contexts, with indications of the tools used to evaluate the performance, the metrics, the benefits, and the shortcomings. The papers^53–58^ referenced on edge caching, placements of functions, and their scheduling without servers, offloading schemes, and autoscaling are web-based and deal with a variety of topics, but very few of them integrate fault tolerance or fault-specific recovery approaches. In contrast, the conceived HGPAFT framework indicates multi-objective optimization, real-time fault management, and DT-aware task reallocation as being exhaustive yet CPU-intensive with an overhead, as it undergoes continuous operations and is processed by a hybrid metaheuristic.

## System architecture and problem definition

To solve the research gap in the development of the Adaptive Fault Tolerance Mechanism for High Availability of Digital Twins in Distributed Edge Computing System, a set of components for failure detection, resource management and digital twin migration should be implemented as a part of the system model and framework and work in real time. Here’s a detailed system model coupled with a framework to support this research problem.

### System model

**Fig. 2:**
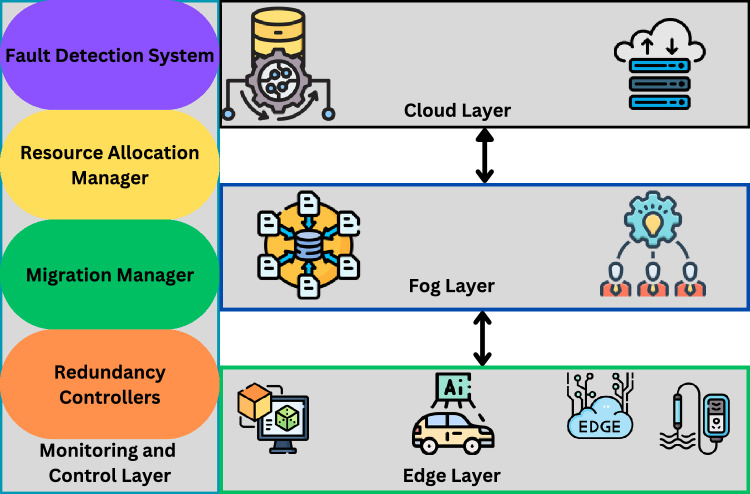
System Model.

The proposed system model given in Fig. 2 combines four layers that work together to ensure the continuity and performance of digital twins in the edge computing context. These layers depict the layered structure of edge, fog and cloud computing that supports to adaptive fault tolerance.

*Edge Layer (Edge Nodes)* The various component of Edge layer are digital twins mirrors of physical assets, non-centralized IoT sensors, and actuators, and edge computing nodes. Handle real time data, update the principals and act locally. This layer is most susceptible to failures, such as; network connection problems, hardware failures among others; it therefore must always be monitored for failure tolerance.

*Fog Layer (Regional Aggregators)*Fog layer has fog nodes, regional data aggregator nodes. Situated between the edge nodes and the cloud, it perform data collection, data forwarding and triggering of resource sharing of edge nodes in case of faults. Fog nodes, therefore, store local copies of the digital twins and help in instance migration when required.

*Cloud Layer* Cloud Layer: It has long-term repository, cloud storage center and large-scale computation service. The role of cloud layer is to standby, support big data processing, and serve as a resource for potentially complicated failure recovery processes. It will be retrieved from local recovery at the edge or fog layer and in the last instance from the cloud.

*Monitoring and Control Layer* It contains Fault detection systems, resource allocation manager, migration manager, redundancy controllers. This layer pervasively assess the readiness of edge nodes, anticipate failures, re-balance loads and trigger digital twin movements. This layer can reside in both the edge and fog layers but can be synchronized using the cloud.

### Framework for adaptive fault tolerance

The proposed framework is divided into four key modules:Monitoring and failure detection, Resource allocation and reallocation, Digital twin migration, Recovery and redundancy management. All the modules are designed to work in conjoint to make the system highly available.

1. *Monitoring and faliure detection module* Monitoring and failure detection module is specially developed to track down possible node failure early on and in realtime. This is achieved by using permanent checks on other important health parameters like CPU usage, memory availability, network latency and error rates in order to identify early warning signs that may precipitate a problem. The system employed by the current solution uses ML algorithms to predict node crashes based on past data and patterns characteristic of failures. In the event that a potential failure is identified, the module immediately notifies the resource allocation and migration modules so that steps necessary to avoid failure and ensure that the best performance is achieved can be implemented.
2. *Resource allocation and reallocation Model* The objective is to dynamically migrate the computations and load from the failed node to the other functional nodes present in the edges. Firstly digital twins are deployed across edge nodes according to the requirements in terms of resource that need to be fulfilled and availability of computing resources in the edges nodes so as to ensure minimal latency and optimal power consumption. After failure identification, the dynamic reallocation module reallocates tasks and resources from the failed node to the edge or fog nodes while ensuring that they do not overload any node. Load balancing algorithms such as Round-Robin, Min-Min, or Max-Min prevent the distribution of loads in a skewed manner and reduce service downtime during the process, whereas energy-based algorithms for load balancing address power considerations and battery life during reallocation.
3. *Digital twin migration model* The objective is to safely and efficiently transfer individual instances of digital twins from one edge node to another in the event of node failure. Digital twins stay real-time aligned with their physical counterparts, and before migration, the current state of the digital twin, such as data, tasks, and operational status, is captured and saved for future continuation without the loss of data. Packaged in lightweight containers like Docker, digital twins can migrate efficiently between nodes, abstracting hardware differences. A cold or hot standby replica can be kept at fog nodes, which can be migrated, or a new replica can be created at a healthy edge node, depending upon the degree of fault. To reduce the service interruption, nearest neighbor strategies are used for the selection of the target node, considering the best time for migration by analyzing the network status.
4. *Recovery and redundancy management Module* The objective here is to facilitate recovery from faults in the system and to guarantee high availability by managing on redundancy as well as fast recovery techniques. Check-pointing is anticipatory, and it periodically backs up copies of the digital twin instances to immune FOG or CLOUD nodes, and a copy can be restored from the latest snapshot in case the node crashes. Redundancy strategies always keep hot standby twins at the edge layer and backup replicas at the fog layer, and it is flexible to switch redundancy based on twin value and risk probability. The general workflow of the recovery workflow starts with a failure detection by the monitoring module, the subsequent task reallocation in healthy nodes, the migration of twins if necessary, and the state recovery starting from the last checkpoint to reduce data loss as much as possible. This approach ensures quick resumption of operations, thus protecting service availability.

### Framework workflow

1. Normal Operation: The system provides digital twin simulations on distributed edge nodes with on-going health checks.
2. Fault Detection: Crashing node monitoring module identifies that a node may fail based on deviations in system metrics (CPU usage, memory, network delay, etc.).
3. Alert and Pre-Failure Migration: If a failure is detected, the resource allocation module triggers a reallocation of tasks to other close edge nodes; the digital twin instance shifts to a hot node.
4. Node Failure: In case of a break or failure of an edge node, the redundancy module becomes active and pulls the updated digital twin from a fog or cloud replica.
5. Resource Recovery: After failure recovery, the system reorients resources; depending on the type of computation required, it may switch the digital twin back to the edge node for continual computations.

**Fig. 3:**
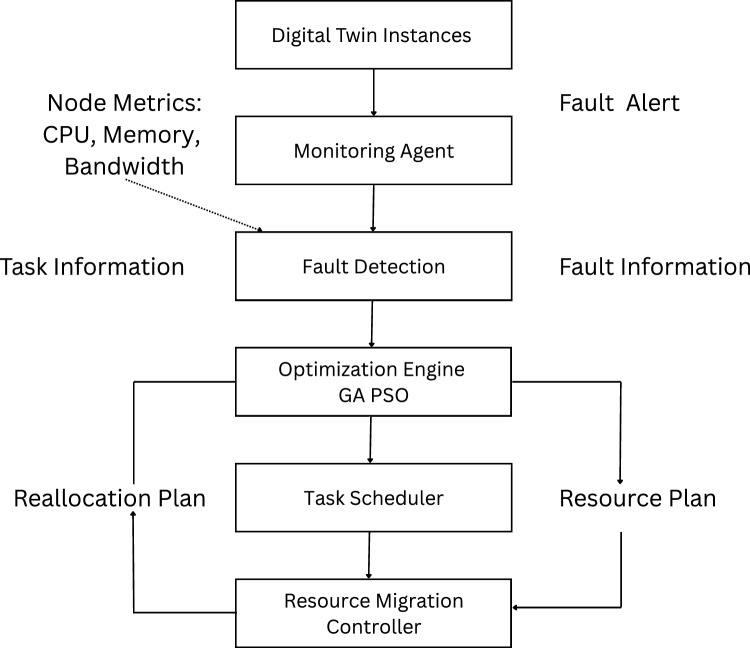
System level interaction of components of HGPAFT framework.

Figure 3 shows the flow of the monitoring, fault detection, optimization, scheduling and resource reallocation processes and internal component interaction within the HGPAFT framework. Digital Twin instances produce live node measurements- including CPU load, memory utilization and bandwidth-, that the Monitoring Agent gathers. These measures as well as task-specific measurements are examined by the Fault Detection module in order to enable detecting anomalies or failures, and to distribute such fault alerts along with diagnostic information to the Optimization Engine. A hybrid Genetic Algorithm and Particle Swarm Optimization (GA-PSO) runs this engine, and calculates optimal task reallocation and resource usage plans. Based on these outputs, the Task Scheduler develops reallocation plans, which will eventually be implemented by the Resource Migration Controller, posing minimal service interference and high availability of Digital Twin services deployed in distributed edge edge settings.

Some of the benefits expected from this system are as follows: Advanced digital twin functions should not be interrupted frequently; otherwise, it will not be viable to maintain up-to-date performance. The system should be able to scale across multiple numbers of edge and fog nodes and depending on the loads and fault conditions on the network. Also, it focuses on energy management, where the fault tolerance is weighed against energy usage to help the whole edge network run efficiently in terms of energy consumption, while keeping up the best possible operating performance.

### Problem definition

When working in a distributed environment of the edge computing and utilizing digital twins, it is essential to guarantee high availability and reliability of the system, including handling faults. Digital twins, which replicate physical entities and processes, must remain operational and accurately synchronized to provide real-time insights and control. Edge computing being a distributed architecture, nodes may undergo failures that result from equipment breakdowns, communication breakdowns, or depleted resources. The issue is to be able to deal with such failures without compromising the overall system’s performance.The problem can be formally defined as follows:

The goal is to propose an adaptive fault tolerance approach for achieving high availability and reliability of the digital twin in the distributed edge computing systems to maximize the recovery probability to quickly bring the digital twin to an operational state, to minimize reallocation and migration costs to reduce the overhead for reallocating resources or migrating digital twin instances among edge nodes, and to minimize the energy consumption while load balancing for an efficient distribution of the work load across the edge nodes.The above problem has following constraints

1. *Resource Availability* In any of the edge nodes, there are limited resources such as CPU, memory and available bandwidth. It must also adapt the reallocation of the tasks or migrate digital twin copies within these constraints.
2. *Fault Detection Time* The designed system should be capable of immediately identifying faults or within an acceptable period after which recovery actions should be taken.
3. *Latency* Transferring a digital twin during an operation should be done in such a way that it will not cause delay primarily in real-time settings.
4. *Energy Efficiency* When performing fault tolerance actions for example, reallocation or migration the system should minimize energy consumption.
5. *Migration Costs* Digital twins moving from one node to another also require communications and recourse, hence should be as less as possible to allow efficiency in the system.
6. *Recovery Time* The time also taken in order to achieve a system state whereby the normal flow of service is achieved in the case of a fault occurrence should also be kept to as low as is possible.

### Mathematical formulation

**Table 2:** Notations used in the model.

Notation	Description
$E = \{e_1, e_2, \dots , e_n\}$	Set of edge nodes
$F = \{f_1, f_2, \dots , f_m\}$	Set of fog nodes
*C*	Centralized cloud node
$T = \{t_1, t_2, \dots , t_k\}$	Set of digital twin instances
$R(e_i)$	Available resources (CPU, memory, bandwidth) on edge node $e_i$
$L(e_i, f_j)$	Latency between edge node $e_i$ and fog node $f_j$
$D(e_i)$	Demand of digital twin instance $t_i$ running on edge node $e_i$ (in terms of resource consumption)
$\alpha$	Probability of edge node failure
$\beta$	Resource reallocation factor
$\gamma$	Migration cost
$M(t_i, e_j)$	Migration of digital twin $t_i$ to edge node $e_j$
$P(t_i)$	Probability of successful restoration of digital twin $t_i$ after node failure
$Q(t_i)$	State of digital twin $t_i$ (0 for failed, 1 for active)
$C_m$	Migration cost (in terms of network bandwidth and state transfer time)
$E_c$	Energy consumption of edge nodes
$R_f(f_j)$	Resources of fog node $f_j$

We aim to detect edge node failures using a predictive model that monitors key system metrics (CPU, memory, bandwidth) and predicts anomalies.The Table 2 represents all symbols and their descriptions used in the mathematical formulation. Let $X_i$ represent the system metric vector at time *t* for node $e_i$. Detect potential node failures using a predictive model based on system metrics (CPU, memory, bandwidth). Let $X_i(t)$ represent the system metrics of node $e_i$ at time *t*, such as CPU utilization, memory usage, and bandwidth. Failure Probability $\alpha _i(t)$ is defined as follows by Eq. 1:

1 $$\begin{aligned} \alpha _i(t) = f(X_i(t)) \end{aligned}$$

where $f(\cdot )$ is an anomaly detection function based on historical and real-time metrics $X_i(t)$ = { $\text {CPU}_i(t)$, $\text {Memory}_i(t)$, $\text {Bandwidth}_i(t)$ } Condition for failure detection is as follows in Eq. 2:

2 $$\begin{aligned} \alpha _i(t) \ge \alpha _{\text {th}} \end{aligned}$$

If the probability of failure $\alpha _i(t)$ exceeds a predefined threshold $\alpha _{\text {th}}$, the system triggers resource reallocation and migration.

When a failure is detected at an edge node $e_i$, we need to reallocate its tasks $t_i$ to another edge node or fog node with sufficient resources. The objective is to minimize the overall resource reallocation cost $\beta _i$ while satisfying the resource requirements, given by Eq. 3.

3 $$\begin{aligned} \min \sum _{i=1}^{k} \beta _i \cdot D(t_i) \end{aligned}$$

where $\beta _i$ is the reallocation factor, and $D(t_i)$ represents the resource demand of digital twin $t_i$. The resource constraint is defined as follows by Eq. 4:

4 $$\begin{aligned} \sum _{i=1}^{k} D(t_i) \le R(e_j) \quad \forall e_j \in E \end{aligned}$$

where $R(e_j)$ is the available resource on edge node $e_j$. Latency constraint is represented as follows by Eq. 5

5 $$\begin{aligned} L(e_i, f_j) \le L_{\text {max}} \quad \forall f_j \in F \end{aligned}$$

where $L_{\text {max}}$ is the maximum allowable latency for digital twin operations.

If the edge node fails or is predicted to fail, digital twin migration ensures continuity by transferring the twin’s state to another node. The objective is to minimize the migration cost $C_m$, which includes state transfer time and bandwidth consumption given in Eq. 6.

6 $$\begin{aligned} C_m =\min \sum _{i=1}^{k} \gamma _i \cdot M(t_i, e_j) \end{aligned}$$

where $\gamma _i$ is the migration cost for digital twin $t_i$, and $M(t_i, e_j)$ represents the decision to migrate twin $t_i$ to node $e_j$. Resource constraint for migration can be represented as in Eq. 7

7 $$\begin{aligned} D(t_i) \le R(e_j) \quad \forall e_j \in E \cup F \end{aligned}$$

ensuring the target node $e_j$ has sufficient resources for the digital twin’s demands. The bound on migration cost is set as in Eq. 8

8 $$\begin{aligned} C_m(t_i) \le C_{\text {max}} \end{aligned}$$

where $C_{\text {max}}$ is the maximum allowable migration cost in terms of network bandwidth and state transfer time.

To improve fault tolerance, redundancy–maintaining backup replicas—is implemented. In case of node failure, recovery is initiated from the fog or cloud layer. The objective is to ensure fault tolerance by leveraging redundancy and backup recovery mechanisms in fog and cloud nodes. The recovery probability $P(t_i)$ of restoring a twin $t_i$ is defined as follows by Eq. 9:

9 $$\begin{aligned} P(t_i) = 1 - \prod _{j \in F \cup C} \left( 1 - \frac{D(t_i)}{R_f(f_j)} \right) \end{aligned}$$

where $P(t_i)$ represents the probability of successfully restoring the digital twin $t_i$ after a failure. $R_f(f_j)$ is the available resource at fog node $f_j$, and $D(t_i)$ is the demand of the digital twin. Recovery Time Constraint is defined as by the Eq. 10

10 $$\begin{aligned} T_{\text {recovery}} \le T_{\text {max}} \end{aligned}$$

where $T_{\text {max}}$ is the maximum allowable recovery time to bring the digital twin back online. The resource constraint for recovery is represented as by Eq. 11

11 $$\begin{aligned} D(t_i) \le R_f(f_j) \quad \forall f_j \in F \cup C \end{aligned}$$

ensuring there are sufficient resources in the fog or cloud to restore the twin.

Since edge computing environments are resource-constrained, energy consumption is a key factor, and we need to balance fault tolerance and energy efficiency. The objective is to minimize the total energy consumption of the system is given by Eq. 12:

12 $$\begin{aligned} \min E_c = \sum _{i=1}^{n} P(e_i) \cdot E(e_i) \end{aligned}$$

where $P(e_i)$ represents the operational state of node $e_i$ (1 if active, 0 if idle), and $E(e_i)$ is the energy consumption of node $e_i$. The energy budget constraint is defined by Eq. 13:

13 $$\begin{aligned} E_c \le E_{\text {budget}} \end{aligned}$$

where $E_{\text {budget}}$ is the maximum allowable energy consumption in the system.

The complete optimization problem that integrates all five models is given by Eq. 14 and constraints:

14 $$\begin{aligned} \begin{aligned} \text {min} \quad&\left( \sum _{i=1}^{k} \beta _i \cdot D(t_i) + \sum _{i=1}^{k} \gamma _i \cdot M(t_i, e_j) + \sum _{i=1}^{n} P(e_i) \cdot E(e_i) \right) \end{aligned} \end{aligned}$$

subject to:

Resource constraints

$$D(t_i) \le R(e_j) \quad \forall e_j \in E \cup F$$

Latency constraints

$$L(e_i, f_j) \le L_{\text {max}}$$

Migration cost constraints

$$C_m(t_i) \le C_{\text {max}}$$

Recovery probability

$$P(t_i) \ge P_{\text {min}}$$

Energy consumption constraint

$$E_c \le E_{\text {budget}}$$

This optimization problem ensures that the system is resilient to failures while minimizing costs, maintaining performance, and staying energy efficient.

## HGPAFT: hybrid genetic-PSO for adaptive fault tolerance

In order to address the efficiency of solving the identified problem of Adaptive Fault Tolerance Mechanisms for Digital Twins in Edge Computing, it is required to have an algorithm capable of considering several objectives at once: minimization of reallocation costs, optimization of migration decisions, high probability of recovery, and minimizing energy consumption. Given that this entails a solution to a Multi-objective optimization problem in a dynamic and distributed environment, In this paper we propose to design a Hybrid Multi-objective Metaheuristic Algorithm that uses both Genetic Algorithms and Particle Swarm Optimization in addition to a Dynamic Fault Detection Module. This approach is known as the Hybrid Genetic-PSO for Adaptive Fault Tolerance (HGPAFT) algorithm and can accommodate to the strict application of fault tolerance, resource allocation and the complexity of the overall system system. Here’s how the algorithm works:

This comprises a dynamic fault detection module for real-time node failure risk assessment based on anomaly detection analysis of system parameters inclusive of the CPU, memory, and bandwidth. After a fault has been identified or projected, the algorithm calls for resource redistribution or targets the digital twin. This paper applies a combined GA and PSO, where GA is used for a global search, as opposed to PSO, used for an efficient local search. The objective functions thus look to minimize the cost and time associated with the reallocation, within the rack, of the migrated storage; maximize the probability of recovery in the event of failure; and minimize energy consumption. Each solution or a collection of node-task mapping or a combination of node and task is then ranked based on a four-objective fitness function.

### Steps

1. Initialization Phase

Step 1.1: Initialize a population of candidate solutions. Each solution is a potential task-node mapping and resource allocation plan.

Step 1.2: For each candidate solution, calculate the fault probability $\alpha _i(t)$ using real-time monitoring of system metrics.

Step 1.3: Initialize velocity and position for PSO particles, and generate the initial population for GA with random crossover and mutation.

2. Fitness Function

For each candidate solution (individual or particle), the multi-objective fitness function *F* is calculated by Eq. 15:

15 $$\begin{aligned} \begin{aligned} F =&\;w_1 \cdot \text {Reallocation Cost} + w_2 \cdot \text {Migration Cost} + w_3 \cdot \\ &\text {Energy Consumption} - w_4 \cdot \text {Recovery Probability} \end{aligned} \end{aligned}$$

where $w_1, w_2, w_3, w_4$ are the weights assigned to different objectives.

Reallocation cost is calculated by Eq. 16 based on the number of tasks reallocated and the resource usage:

16 $$\begin{aligned} C_{\text {realloc}} = \sum _{i=1}^{k} \beta _i \cdot D(t_i) \end{aligned}$$

Migration cost given in Eq. 17 includes the state transfer cost of migrating digital twins from one node to another:

17 $$\begin{aligned} C_{\text {mig}} = \sum _{i=1}^{k} \gamma _i \cdot M(t_i, e_j) \end{aligned}$$

Total energy consumed by the system is defined in Eq. 18 :

18 $$\begin{aligned} E_{\text {cons}} = \sum _{i=1}^{n} P(e_i) \cdot E(e_i) \end{aligned}$$

Recovery probability given in Eq. 19 measures the likelihood of successful recovery of a digital twin after node failure:

19 $$\begin{aligned} P_{\text {recov}}(t_i) = 1 - \prod _{e_j \in F \cup C} \left( 1 - \frac{R_f(f_j)}{D(t_i)} \right) \end{aligned}$$

3. GA Operations

Step 3.1: Perform Selection: Choose parent solutions based on their fitness values using a tournament selection or roulette wheel method.

Step 3.2: Apply Crossover: Exchange portions of task-node mappings between parent solutions to generate offspring.

Step 3.3: Apply Mutation: Randomly change task assignments or resources in the solutions to maintain diversity in the population.

4. PSO Operations

Step 4.1: Equations 20 and 21 is used to update each particle’s velocity and position according to the PSO update rules:

20 $$\begin{aligned} v_i(t+1) = w \cdot v_i(t) + c_1 \cdot r_1 \cdot (p_i - x_i(t)) + c_2 \cdot r_2 \cdot (g_i - x_i(t)) \end{aligned}$$

21 $$\begin{aligned} x_i(t+1) = x_i(t) + v_i(t+1) \end{aligned}$$

where $v_i$ is the velocity, $x_i$ is the position, $p_i$ is the best local solution, and $g_i$ is the global best solution.

Step 4.2: Evaluate the fitness of the new particle positions using the same multi-objective fitness function.

5. Hybrid GA-PSO Iteration

Step 5.1: Combine the new populations from GA and PSO into one. Evaluate the fitness of all solutions and rank them.

Step 5.2: Apply a Pareto-front ranking to maintain a diverse set of non-dominated solutions across all objectives.

Step 5.3: Repeat the GA-PSO process for a predefined number of iterations or until convergence criteria are met.

6. Fault Tolerance and Decision Execution

Step 6.1: Once the best solution is found, execute the corresponding task reallocation or migration plan.

Step 6.2: If failure occurs, initiate recovery using the checkpoint mechanism, and migrate digital twins to fog or cloud nodes.

### HGPAFT algorithm

**Algorithm 1:**
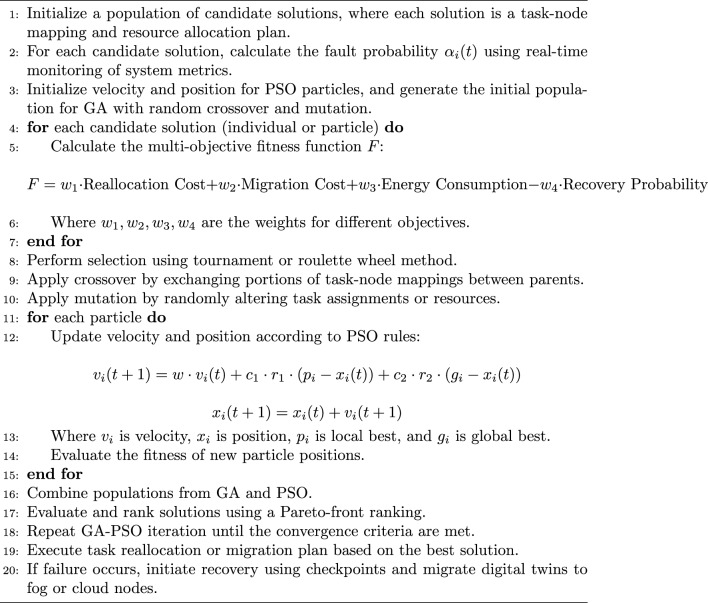
HGPAFT Algorithm.

**Algorithm 2:**
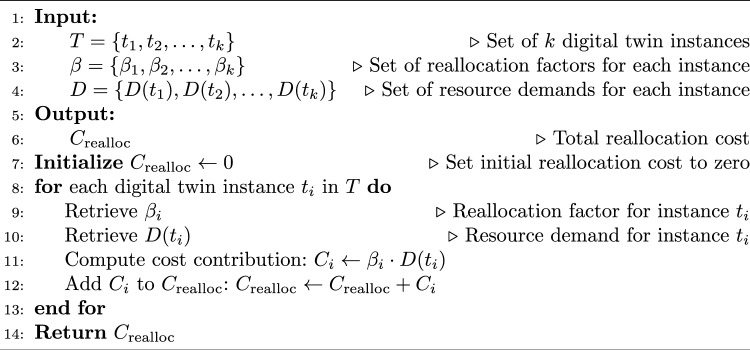
Reallocation Cost Calculation.

**Algorithm 3:**
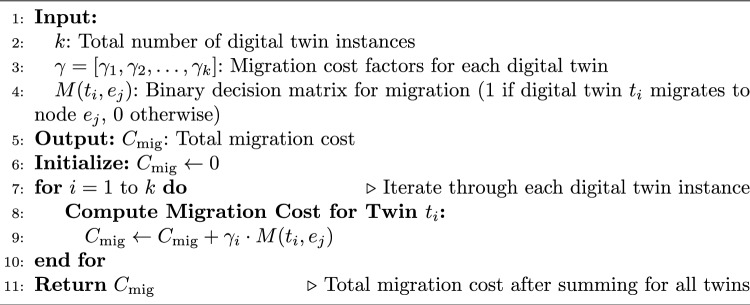
Compute Migration Cost $C_{\text {mig}}$.

**Algorithm 4:**
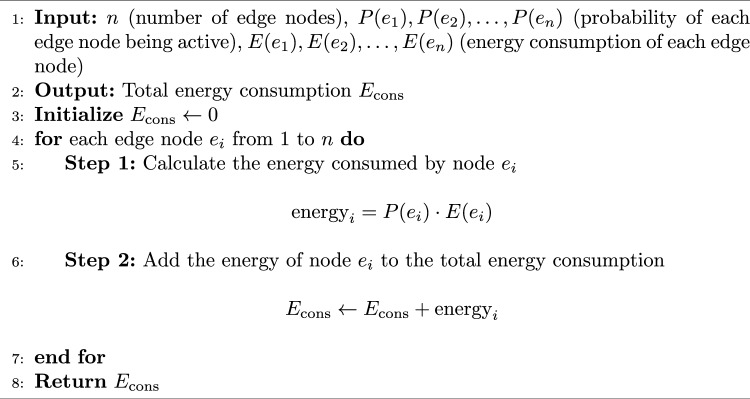
Energy Consumption Calculation.

**Algorithm 5:**
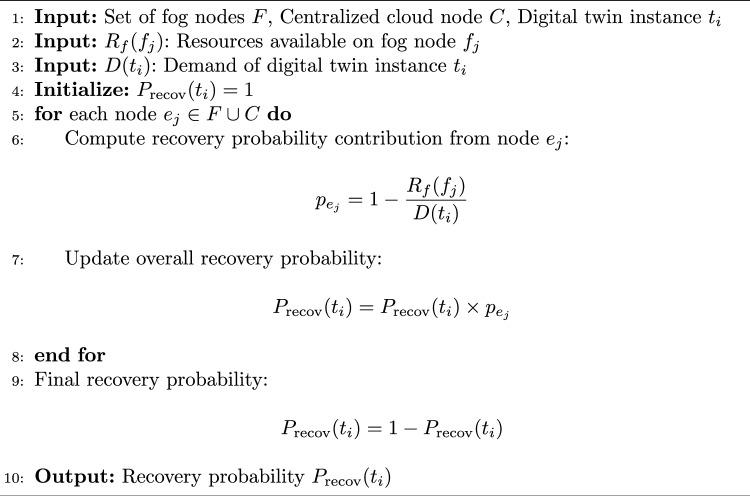
Computation of Recovery Probability $P_{\text {recov}}(t_i)$.

The pseudo-code of HGPAFT algorithm is mentioned in Algorithm 1. It is the primary hybrid algorithm of optimization using a combination of Genetic Algorithm (GA) and Particle Swarms Optimization (PSO) to manage adaptive fault tolerance. It starts with an initial population of solutions (task-node mappings), performs a multi-objective evaluation based on an objective (reallocation cost, migration cost, energy consumption, recovery probability) and iteratively optimizes the solutions based on the GA and PSO operations. The most optimal solution found at the end is employed to perform the fault-tolerant activity (digital twin migration or task reallocation). The process of calculating reallocation cost is described in Algorithm 2. The algorithm calculates reallocation cost on the complete task reallocation after either predicted or actual node failure. It scales the resource demand of that task by that digital twin reallocation factor and aggregates that by each digital twin to yield the total cost of reallocation. The steps of finding migration cost is mentioned in Algorithm 3. This algorithm determines the cost of migration of digital twins to healthy nodes. On each twin it tests the occurrence of migration (through a binary migration matrix) and multiplies the migration factor with this decision to a given level and then adds the result to have the total migration cost. Energy consumption calculation are described in Algorithm 4, This algorithm considers the overall energy of all the edge nodes. It also multiples the operational condition (active or idle) of individual nodes, with their specific energy consumption and collects that to obtain an estimate of the system energy consumption of the entire system during existing fault productive operation. The computation of recovery probability is given by Algorithm 5, This algorithm calculates the likelihood of effectively recovering a digital twin by consuming resources within fog and cloud nodes. It sums up the resilience of each backup node and uses a formula to come up with the likelihood that the digital twin is recovered when a failure takes place.

The HGPAFT algorithm implementation starts with the initialization of the population of candidate solutions, the one where every solution is the code representing a possible solution to mapping the tasks to nodes and solving the problem of assigning resources to pieces. Real-time node metrics (CPU utilization, memory availability and bandwidth) are then continuously monitored by the system to calculate fault probabilities $\alpha _i(t)$ of every node. Using these probabilities, the algorithm uses Genetic Algorithm (GA) operations of selection, crossover and mutation to explore the global search space, and Particle Swarm Optimization (PSO) operations which optimize the solution through updating of velocity and position with the help of individual best and global bests. Next and briefly, candidate solutions are scored via a fitness objective (as seen in Equation 15) that considers reallocation, migration, energy expenditure as well as recovery likelihood. The solutions of best solutions of GA and PSO are combined and ordered based on Pareto-front dominance to maintain a population of the variety of solutions that are optimum. At last, according to the first-rated solution, the system realizes the respective task reallocation or digital twin migration plan and marks the consequent system state.

### Computational overhead and time complexity analysis

Computational complexity of the proposed HGPAFT is discussed with relation to the key elements, i.e., hybrid GA-PSO optimization, monitoring, fault detection, and task migration. The time complexity of Algorithm 1 (Initialization) is $\mathcal {O}(N \cdot M)$ since $P$ chromosomes are generated with the length proportional to the number of the tasks, $N$. Algorithm 2 (Fault Probability Estimation) calculates the fault probability$\alpha _i(t)$ on each node based on monitored resource values, and has complexity of $\mathcal {O}(M)$ in each cycle of monitoring. The operations that make up algorithm 3 include Algorithm- 3 (GA-PSO Evolution) selection, crossover, mutation, and velocity/position updates of each of the individuals in a fixed number of generations respectively $G$ and each fitness calculation is carried out with migration and cost estimation thus the overall complexity is $\mathcal {O}(G \cdot P \cdot N)$. Pareto-front ranking and migration planning that are used in Algorithm 4 (Task Reallocation) will provide a complexity of $\mathcal {O}(N \cdot \log N + N \cdot M)$. In summary, HGPAFT has a polynomial time complexity in terms of number of tasks and nodes, hence is computationally viable to be used in the practical, medium size distributed edge networks.

## Evaluation and experimental setup

This section describes how the performance of the Adaptive Fault Tolerance Mechanisms for guaranteeing high availability of digital twins in decentralized edge computing systems was assessed. This work was intended to define a real-world-like experiment in a distributed edge computing context, assess performance through different metrics and compare the HGPAFT against benchmark techniques.

### Simulation environment

Hardware and simulation environment resemble actual implementation of edge computing technologies in industry like smart manufacturing frameworks, industrial IoT, and intelligent building and infrastructure monitoring systems. The edge, fog, and cloud configurations, together with injected faults are among the configurations that are common in real-life settings. The environment used to test the fault tolerance mechanism was developed with the intention to closely model the edge computing and digital twin spaces. The hardware and software configuration that builds up the framework is designed to mimic distributed edge computing environments to compare the algorithm’s performance under different conditions.For the edge nodes, the hardware setting was a set of ten distributed edge nodes that were idealized as edge servers or devices. These nodes were particularly limited in terms of CPU, memory, and network capacity to serve as realistic models of the edge computing environments as per the Table 3. Each node was endowed with a finite number of computational resources, memory, and network connections that may be characteristic of edge systems.

**Table 3:** Edge node hardware configuration.

Component	Specification
Processor	Quad-core ARM Cortex-A72 @ 1.5 GHz
Memory	8 GB RAM
Storage	256 GB SSD
Network bandwidth	100 Mbps

The simulation also used a remote cloud infrastructure to illustrate how cloud offloading works should any of the edge nodes fail. The cloud nodes were endowed with far greater computational power compared to the edge nodes so as they were capable of performing tasks preferably offloaded from the edge nodes to enhance system efficiency and stability. Table 4 describe the cloud node hardware configuration.

**Table 4:** Cloud node hardware configuration.

Component	Specification
Processor	16-core Intel Xeon @ 2.5 GHz
Memory	64 GB RAM
Storage	1 TB SSD
Network bandwidth	1 Gbps

The software configuration for the simulation involved using iFogSim as the major simulator for using edge computing as well as simulating interactions between the digital twin and the edge and fog nodes for dynamic simulation of edge and fog environments and integrated fault-tolerance and resource management. MATLAB was used to both implement the Hybrid Genetic-PSO Algorithm and to run the fault tolerance simulations using MATLAB optimization and simulation toolboxes. Docker’s containerization was adopted to develop instances of digital twin using a lightweight virtualization approach; the corresponding DT instances run at every edge node, representing physical objects such as industrial machines or IoT devices, and processing real-time data to synchronize with their physical counterparts. The workload to simulate was generated using EdgeBench which includes object uploads, size and rate of received sensor readings and control messages for IoT devices. A fault injection module was also instantiated using iFogSim’s fault tolerant extension in order to be able to induce failures of different types to be able to control and experiment the type of failure to be induced throughout the experimentation phase.

### Evaluation metrics

In order to provide an in depth analysis of the efficiency of the considered adaptive fault tolerance mechanism, a set of key performance indicators were introduced. These metrics describe system characteristics for keeping the high availability and reliability of digital twins with much less overhead for their fault tolerance.

1. *Downtime* It is defined as the amount of time, in seconds, that a digital twin is offline because of failed edge nodes. Ideally, it’s about avoiding interruption, meaning digital twins are rarely offline or inactive. It was documented every time an edge node failure happened, then the steps taken to revert or the tasks shifted to the next node.
2. *Recovery time* It is the time that is taken before a failure can be identified, and the failed digital twin instance migrated/re-allocated to a healthy node, is instrumental towards provision of service continuity. The goal was to reduce recovery time, defined as the time between the identification of a fault and the restoration of the corresponding digital twin on a new node.
3. *Resource overhead* It encompasses extra computational and memory overheads used in monitoring, fault detection, and migration algorithms for fault tolerance. The goal of the fault tolerance solution is to contribute as little as possible to overhead to ensure that system performance is not greatly affected. Overhead in terms of resources was computed as a ratio of the amount of resources used by the adaptive mechanism to the total system’s usage in the absence of a fault tolerance feature.
4. *Energy Consumption* Energy in Joules can be defined as the number of Joules that the edge nodes utilise in the simulation, especially when being recovered or when migrating digital twins. The goal was to reduce the energy overhead incurred for fault tolerance, particularly in the limited capacity edge systems. Energy awareness simulation was carried out all through the simulation of the environment with the use of energy model of iFogSim which predicts the energy required for computation, communication, and migration.
5. *Task completion rate*The task completion rate (%) it is the percentage of the tasks given to the digital twins out of which are completed including in the presence of faults. The need to maintain a high task completion rate is to be able to sustain the functionality of the system throughout a fault event. This was determined by the number of tasks that were successfully accomplished to the total tasks that had been set during the entire simulation period.
6. *Relocation cost* Reallocation cost in seconds is about the time and resources required for reallocation of tasks and instances of a digital twin to healthy nodes. The goal is to reduce this cost for optimizing the performance of a system while not going over on overhead. It is measured in the sum of the time devoted to migration and rescheduling of tasks due to failures of nodes.

### Real-world fault case studies

In order to test the scale of the proposed HGPAFT framework in real life, we modelled and simulated four typical case studies that represent a common failure situation in the actual distributed edge computing scenario. These scenarios do not only confirm the theoretical constructs of the algorithm but also demonstrate plausible operational perturbations on such levels of industrial automation, smart cities, and distant implementations of the IoT. The Edge Node Failure simulates the failure of one or more edge nodes caused by hardware crash or software errors, as ubiquitous in smart factories or healthcare systems involving resource-limited sensors in harsh environments. A second model, Network Partition, simulates transient disruption of links between edge nodes, e.g. jittery wireless links in remote farms or mining facilities, where link failures were periodically injected to stress fault detection and resource reallocation. The third scenario is Resource Contention or Overload, which reproduces the cases when edge nodes are afloat with a large number of tasks to perform, and this is often the case in the smart traffic control systems during peak hours; the workloads were pre-conditionally enhanced to check how the load balancing in the system responds. Finally, the Cloud-Offloading Failure scenario views the unavailability of the cloud layer temporarily, such as it happens in rural settings or cloud service interruptions, and checks whether the system is able to operate within the edge and fog layers independently. The fault case studies in aggregate evidence the resilience and flexibility of the HGPAFT structure in various, dynamic, and resource-limited environments that are close to real-world edge computing issues. This fault tolerance mechanism was exercised under different fault types to assess how it would handle different faults. The following cases illustrate mishaps normally found in distributed edge learning conditions.

1. *Edge node failure* The simulation scenario that we adopted comprised failures on one or several edge nodes splitting at any time caused by hardware breakdown or network loss. Then random node failures were applied at the different moment in the course of the simulated experiment, and the value of the failed nodes varied from 1 to 3; the frequency of failures was changed from the occasional practice to the constant one. The goal was to assess how efficiently the system identifies node failures and redistributes the digital twins on the other nodes or other cloud resources, thus reducing the time to failure.
2. *Link failure * Network partition or link failures involve a disconnect or breakup between two edge nodes making some sections of the network unavailable thus impacting the communication between the digital twin and the edge server. In the simulation, these network partitioning events were implemented by running a script that would switch off the interconnect link that exists between some of the edge nodes by 10 to 30 seconds. The goal was to try the network faults and examine the abilities of the fault tolerance mechanism in the management of resources reallocation and recovery.
3. *Resource contention/overload*Cognitive overload refers to a state where edge nodes get overwhelmed by tasks they are handling that may lead to suboptimal performance. In the simulation, more load was given to a particular edge node to develop the resemblance of resource congestion where the delay was observed in task handling and the digital twin. The purpose was to evaluate its capability to adjust loads and avoid load localisation from becoming an issue.
4. *Cloud-offloading failure*When using cloud offloading as the backup of resource-limited edge nodes, the unavailability of the cloud and their slow response was also tested. The conducted simulation entailed the blocking of cloud access for a predetermined amount of time which made the system work independently on tasks within the edge layer. The goal was to assess the performance of the adaptive mechanism in failure scenarios of cloud services and to determine its ability to manage tasks in edge networks.

**Table 5:** Simulated fault scenarios and their real-world equivalents.

Fault scenario	Real-world example
Edge Node Failure	Sensor/controller failure in a smart factory or healthcare system
Network Partition	Unstable connectivity in remote agricultural or mining deployments
Resource Contention	Overloaded gateway in smart traffic or surveillance networks
Cloud-Offloading Failure	Cloud unavailability in rural IoT deployments

As Table 5 shows, simulated fault scenarios applied in our experiment and its practical applicability to real-world contexts are mapped. Such mappings strengthen the relevance of the suggested HGPAFT model to different and varied edge computing scenarios of dynamic edge failure and limited resources.

## Results and discussions

The analysis of HGPAFT in various real life fault situations assures its viability in the practical world. Its use in the smart grid, industrial automation, and connected health system applications has been justified by the regular advancements made in recovery time, energy efficiency, and system availability in these areas.

**Fig. 4:**
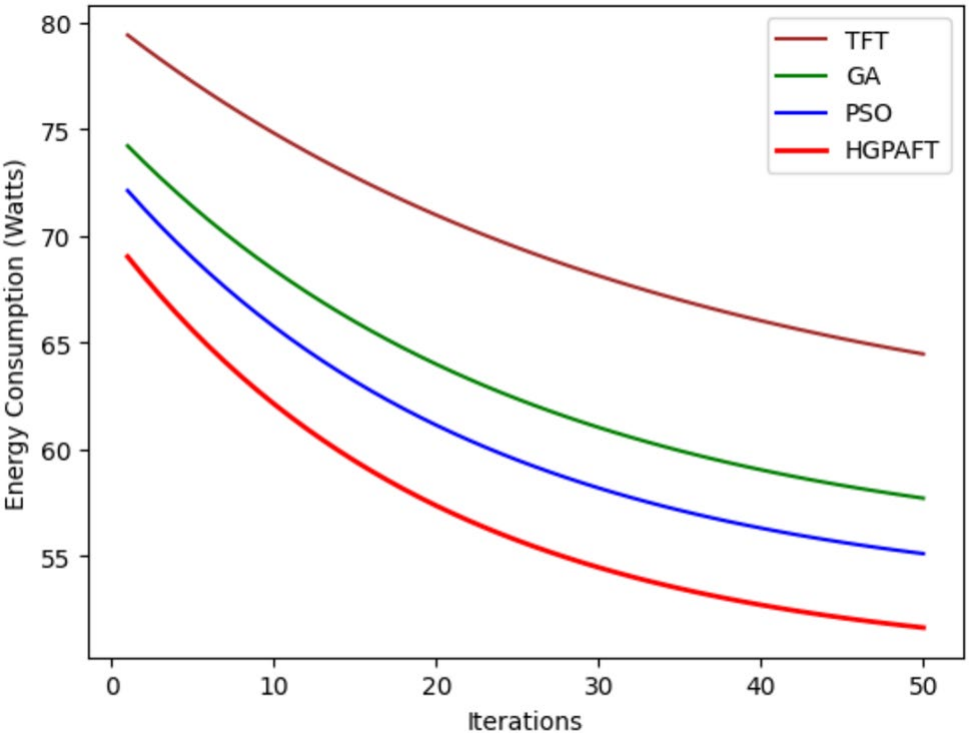
Energy consumption comparison.

The Fig. 4 presents the energy consumption of four algorithms, namely TFT, GA, PSO, and HGPAFT for 50 iterations in Watt. Starting from the initial power of 80 Watts, TFT consumes the most power followed by GA consuming 75W, PSO 70W, and the HGPAFT consuming 65W revealing the efficiency of HGPAFT right from the start. It is established that as the iterations go through all algorithms, energy consumption starts to reduce gradually. The analysis of the 50th iteration shows that TFT still consumes the most energy, which is slightly above 65 Watts, whereas GA and PSO consume about 60 Watt and 57 Watt, respectively. HGPAFT maintains its efficiency advantage through to the end and ends at the lowest energy consumption approximately 55 Watts thus showing how it can optimize resource usage better than the other algorithm. This consistency demonstrates the efficiency of HGPAFT for addressing scavenging and energy optimization in resource-scarce conditions.

**Fig. 5:**
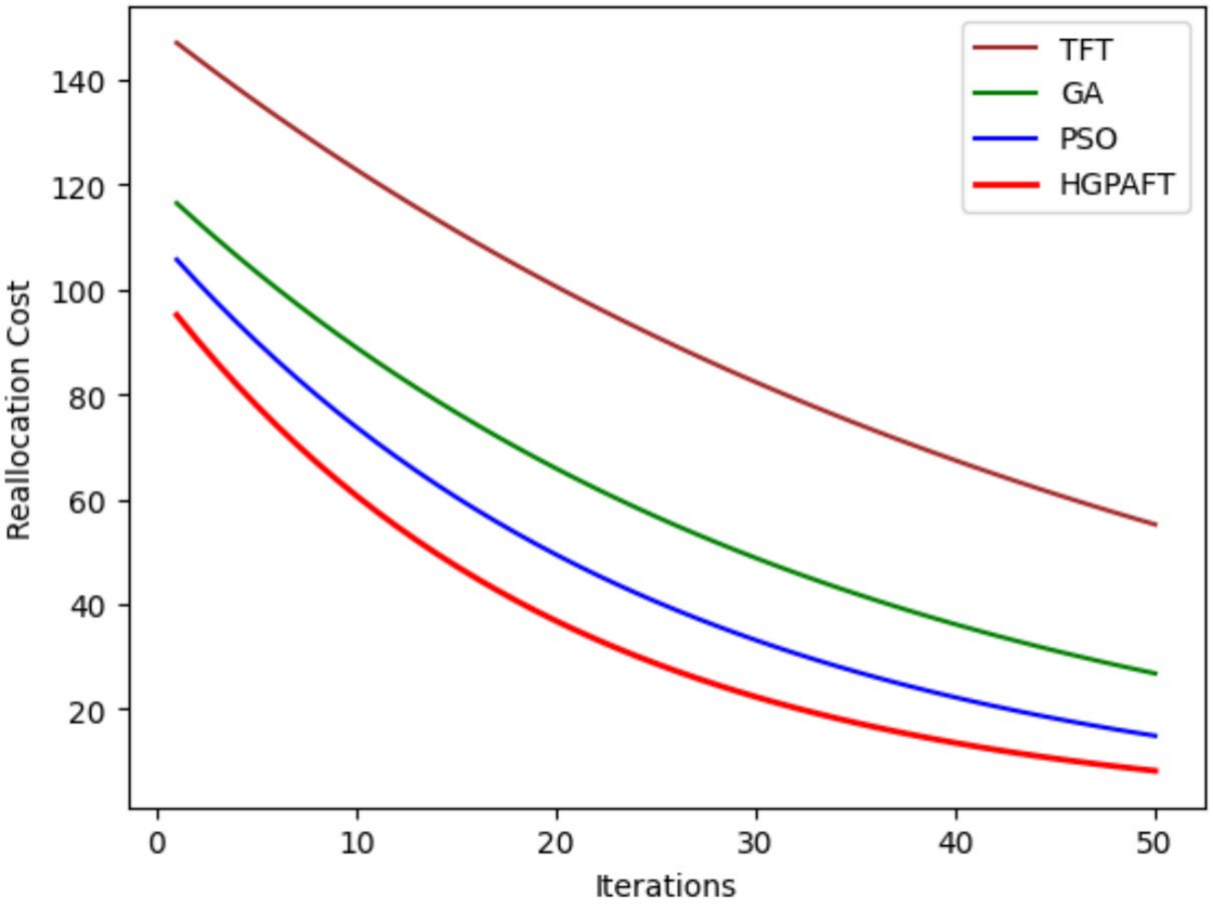
Reallocation cost comparison.

This Fig. 5 represents the reallocation cost variations of four algorithms, namely, TFT, GA, PSO, and HGPAFT, for 50 iterations. Initially, TFT has the highest load reallocation cost of approximately 140 units while GA has a load reallocation cost of 110 units, PSO has a load reallocation cost 100 units and HGPAFT has the least load reallocation cost of 80 units out of all the tested algorithms. Over the iterations, the reallocation cost decreases in all algorithms and stabilize quantitatively. Even after 50 iterations, TFT remains the least effective in comparison with GA and PSO that have a cost of 60 and 50 units, respectively. HGPAFT continues to sustain its excellent performance, with the least reallocation cost of approximately 30 units. This comparison demonstrates that the proposed HGPAFT is superior to the other algorithms in terms of improving the efficiency of task reallocation and enabling the reduction of system overhead in an edge computing context.

**Fig. 6:**
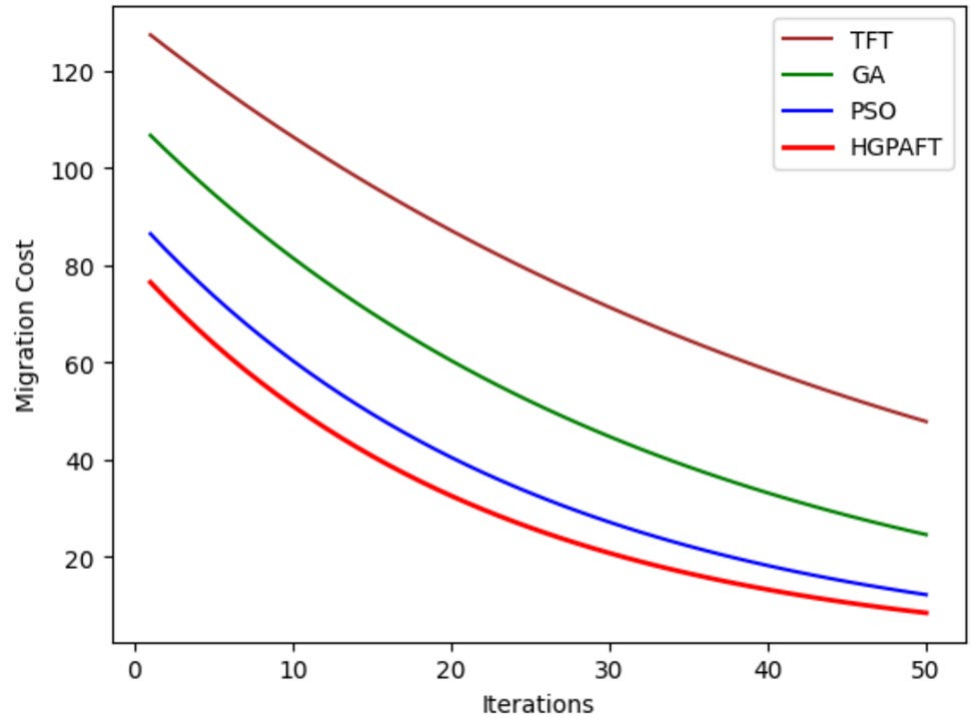
Migration cost comparison.

The Fig. 6 above indicates how the migration cost of four algorithms, namely, TFT, GA, PSO, and HGPAFT, differs across iterations 1 to 50. Initially, the migration cost to TFT is estimated to be at 120 units, while for GA is at 100 units, PSO at 80 units, and the lowest for HGPAFT at 60 units. The graphs for all algorithms show that there is a gradual decline in the migration cost as the iterations increase. Also, by 50th iteration, the TFT is still the most expensive in terms of migration cost with a value of roughly 80 units while the GA and PSO had lesser value of 60 and 40 units respectively. HGPAFT also proves its better efficiency with the least migration cost which is around 20 units by the end of the iteration. This trend indicates that, overall, HGPAFT can enhance migration processes efficiently as it poses lesser overhead and enhances system performance than other algorithms.

**Fig. 7:**
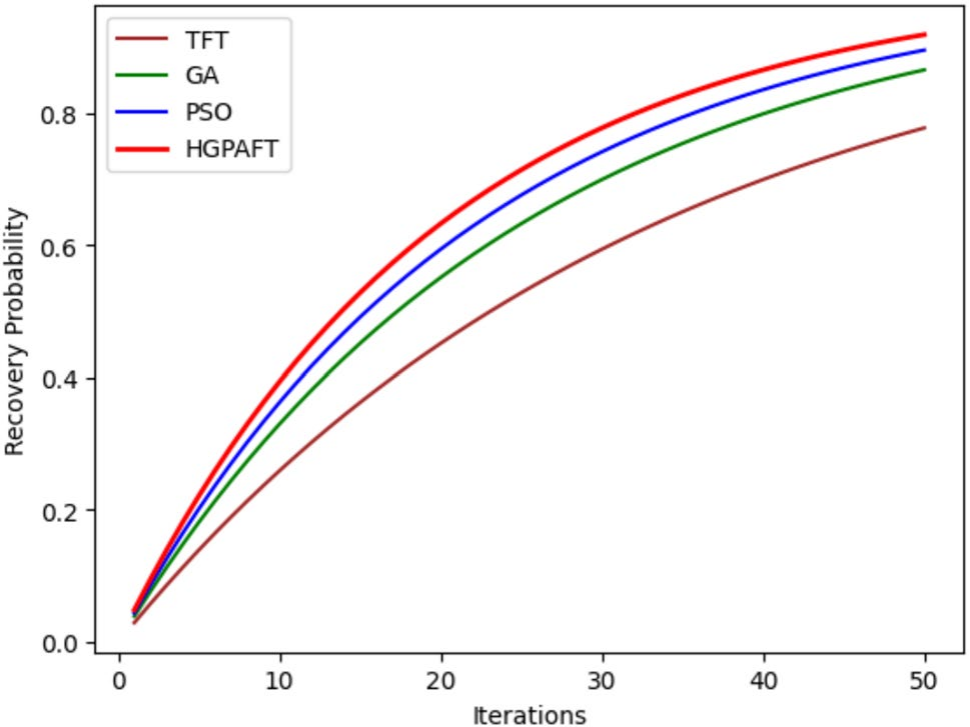
Recovery probability comparison.

Figure 7 shows the probability of recovery in relation to 50 iterations of four algorithms, namely TFT, GA, PSO, and HGPAFT. In the first iteration, each algorithm is initialized with a recovery probability of around 0.0, therefore, poor fault tolerance capacity in the initial phases. In each of the iterations, it can also be identified that the recovery probabilities of all the algorithms have improved over time. After the 50 th iteration, HGPAFT provides the highest recovery probability of closes to 0.9 while PSO and GA are approximately 0.85 and 0.8 respectively. However, in the case of TFT, all players visit the same node and the final obtained utility amounts only to about 0.7. This upward trend demonstrates the increased efficiency of HGPAFT for a system recovery, which proves that the algorithm can ensure reliable edge computing with high reliability over the other algorithms in this comparison.

**Fig. 8:**
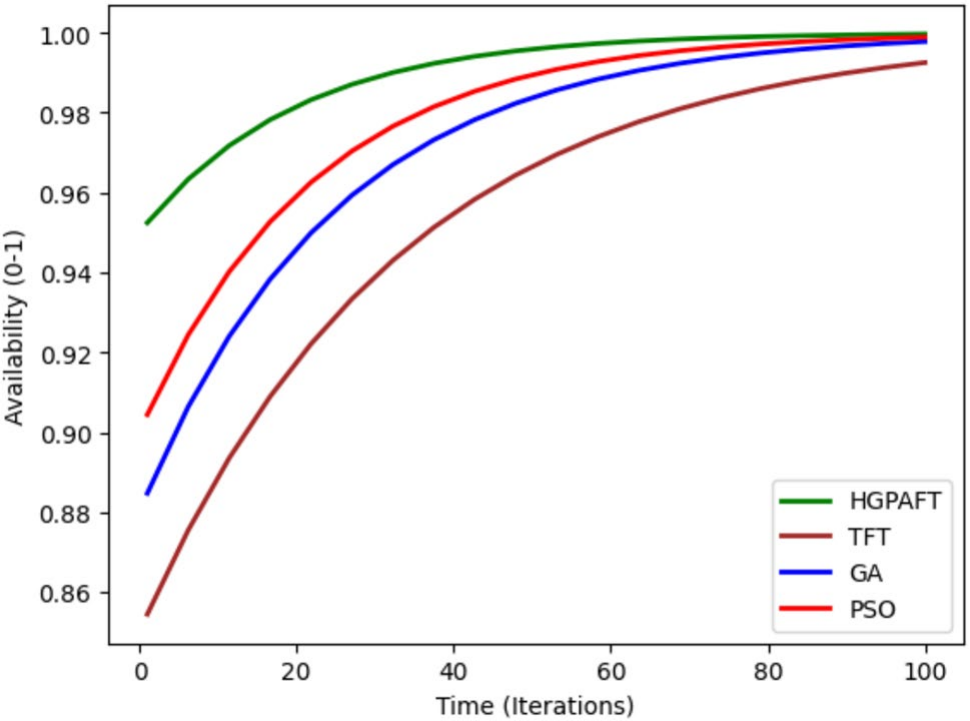
Availability comparison.

Figure 8 shows the availability of four algorithms namely, HGPAFT, TFT, GA and PSO over a maximum of 100 iterations. At the beginning, TFT shows the lowest availability at 0.86 whereas HGPAFT began at 0.95, GA at 0.88 and PSO at 0.9. Over iterations, the availability increases for all algorithms while starting from the same level of one, HGPAFT ends the process having almost unity value by the 100th iteration and thus is better in terms of availability of the whole system. However, TFT converges to a higher accuracy at a slower rate, having a value of about 0.97 while GA and PSO have a value of 0.98 & 0.99, respectively. From this upward trend, it is clear that HGPAFT has always out-performs the other algorithms in retaining near-perfect availability, thus making it the most dependable and stable algorithm among all four.

**Figure 9:**
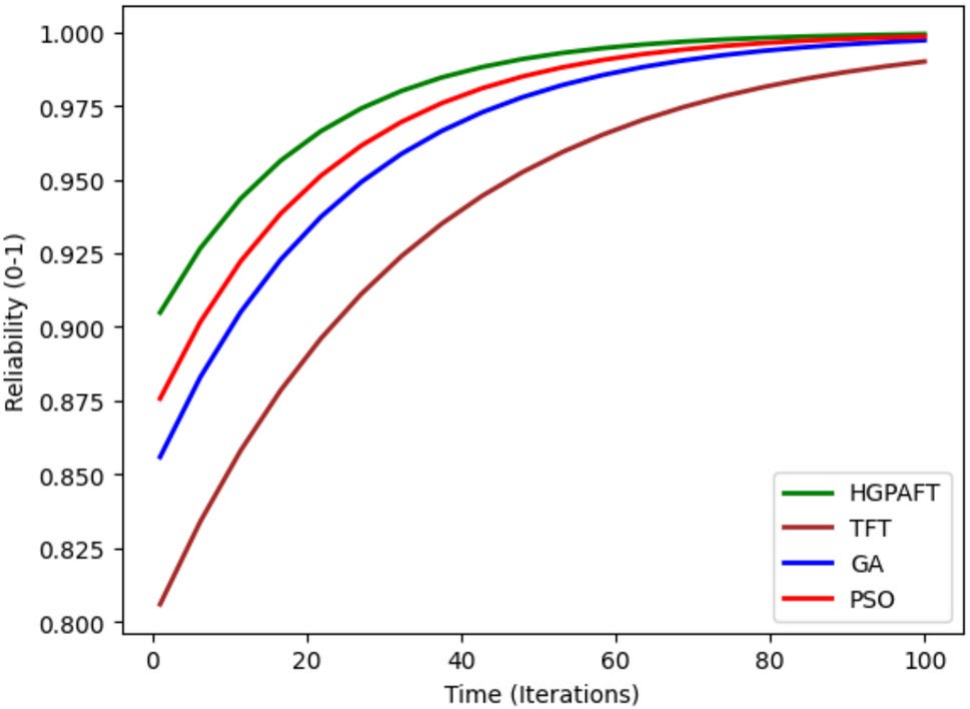
Reliability comparison.

The Fig. 9 shows the reliability comparison of four algorithms to build potential HGPAFT, TFT, GA, and PSO after 100 iterations. First, TFT has the least initial reliability estimated to be around 0.80 suggesting that it offers little guarantee to operational reliability during Faults. All four algorithms employ the reliability measure with HGPAFT starting slightly higher at 0.90, while GA and PSO at 0.85 and 0.87 respectively. Through the iterations, all algorithms remain consistent in their enhancement of reliability across the system. Even by the 100th iteration, the HGPAFT picks the highest reliability marking successility of neared 1.00 which marks the exceptional capability of the fault tolerant system. PSO is next with a reliability estimate of, roughly 0.98, followed by GA which is slightly below at a 0.97, however TFT has a comparatively low reliability estimate of 0.95. These results clearly show that HGPAFT performs significantly better in achieving the objective of system reliability than the other algorithms in large scale and faulty environment.

**Fig. 10:**
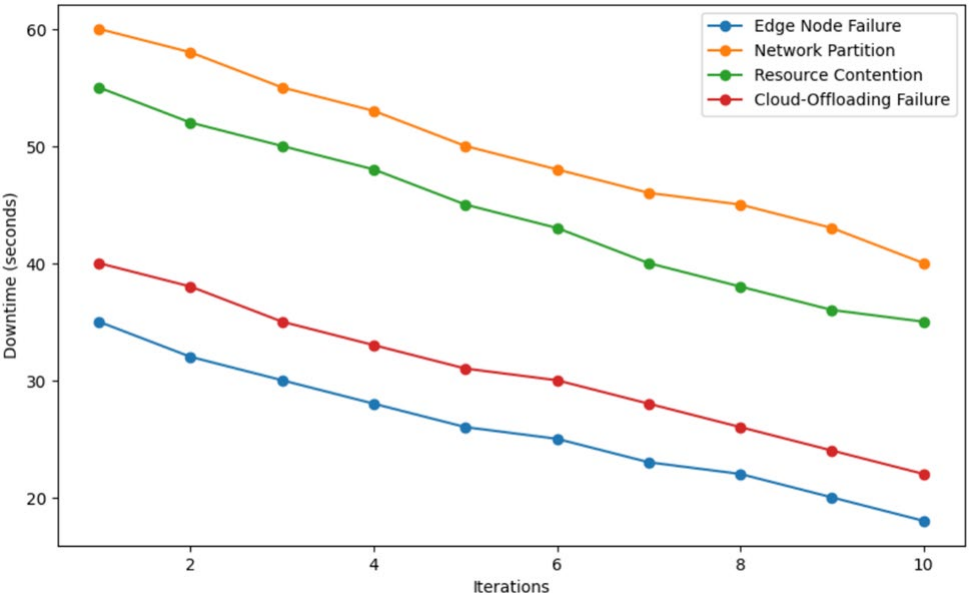
Down time comparison in 4 scenario.

The Fig. 10 shows the downtime of the system in 10 iterations of four categories of failure: Edge Node Failure, Network Partition, Resource Contentions, and Cloud-Offloading Failure, all in seconds. Based on the simulation, Network Partition slows down the system the most with an initial downtime of roughly 60 seconds, seconded by Resource Contention at 55 seconds, and Cloud-Offloading Failure starts at 40 seconds while Edge Node Failure has the least with 30 seconds. For all the cases considered, the downtime steadily declines throughout iterations, and it improves notably even under area changes. Comparing all the iterations 10 the simulation shows that Network Partition is still the highest at 40s for downtime for Resource Contention is at 35s for Cloud-Offloading Failure and Edge Node Failure downtimes are at 25s and 20s respectively. These trends illustrate the fault-tolerance of the system and demonstrate how it reduces the system’s downtime in numerous different fail conditions while also determining that whilst the Edge Node Failure demonstrated the greatest efficiency, the Network Partition was the most inefficient.

**Fig. 11:**
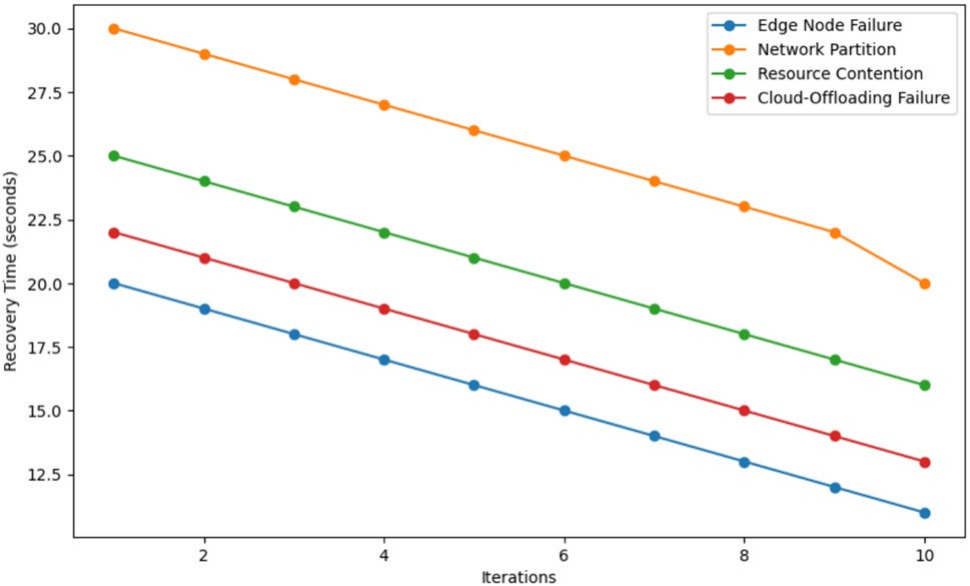
Recovery time comparison in 4 scenario.

This Fig. 11 shows the recovery time characteristics of four failure types: Edge Node Failure, Network Partition, Resource Contention, and Cloud offloading Failure across 10 iterations in seconds. In the actual comparative results for all the scenarios that were investigated, Network Partition has the highest degree of recovery time of about 30 seconds and is succeeded by Resource Contention at 25 sec. On the other hand, Cloud-Offloading Failure considers approximately 20 seconds and lastly, Edge Node Failure at only 15 seconds. For all cases, time to recover experiences a continuous reduction as the number of iterations increases. In the 10th iteration, Network Partition has the longest recovery time of approximately 22 seconds and whereas Resource Contention, Cloud-Offloading Failure, and Edge Node Failure have more decreased to about 15 seconds recovery time, 18 seconds, and 12 seconds respectively. This trend shows how over iterations the system is capable of enhancing recovery mechanisms as Edge Node Failure has the quickest recovery time while Network Partition is the most difficult to recover from.

**Fig. 12:**
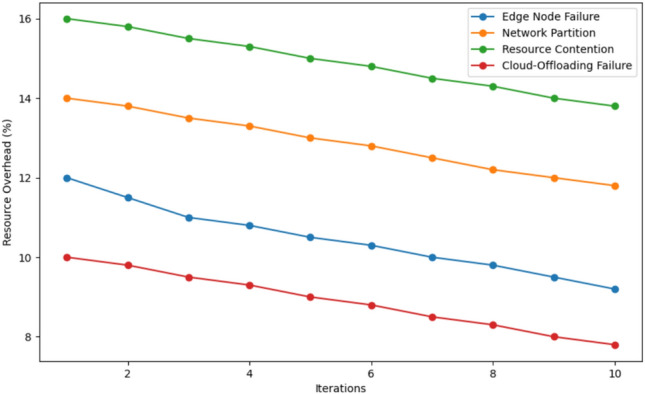
Resource overhead comparison in 4 scenario.

As depicted in this Fig. 12, the percentage of resource overheads in 10 iterations for four failure scenarios including Edge Node Failure, Network Partition, Resource Contention, and Cloud-Offloading Failure, are presented. Subsequently, when comparing the scenarios, Resource Contention has a greater resource overhead at the onset at approximately 16%, Network Partition at 14%, Edge Node Failure at 12%, and Cloud-Offloading Failure with the least at 10%. Across iterations, the resource overhead reduces slowly for all the cases as illustrated in the next section. In the 10th iteration, Resource Contention is still the proposed mechanism with the highest resource overhead which is approximately 14%, the Network Partition, Edge Node Failure, and Cloud-Offloading Failure mechanisms have the overhead of approximately 12%, 10%, and 8%, respectively. This trend shows the internal capability of the system in terms of resource utilization over time and it shows that Cloud-Offloading Failure remains to be the resource with the least overhead on resource usage while Resource Contention remains to be the most difficult resource in terms of optimization.

**Fig. 13:**
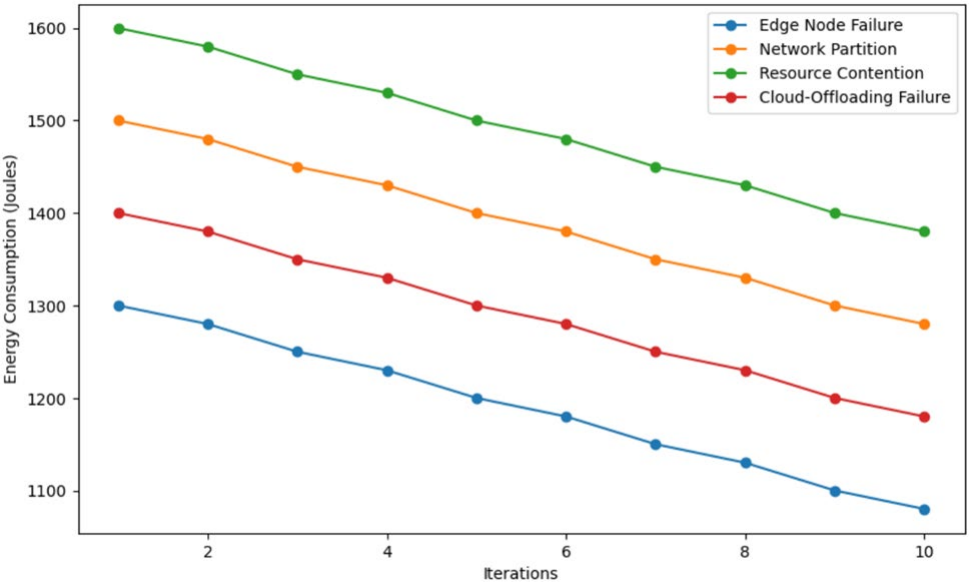
Energy consumption comparison in 4 scenario.

This Fig. 13 illustrates the energy consumption (in Joules) pattern of four failure situations, viz., Edge Node Failure, Network Partition, Resource Contention, and Cloud-Offloading Failure across 10 iterations. Interestingly, Resource Contention consumes the highest energy of approximately 1600 Joules depending on the simulation setup, Next, Network Partition takes the second position with consuing 1500 Joules during the simulation experiment Fourthly, Cloud-Offloading Failure comes third, and finally, Edge Node Failure the least with an energy consumption of around 1300 Joules. It’s also evident that, as iterations go through progressive levels, the energy consumption value decreases for all the scenarios. At the 10th iteration, Resource Contention is still the most dominant component at nearly 1400 Joules while Network Partition, Cloud-Offloading Failure, and Edge Node failure are at about 1350 Joules, 1300 Joules, and 1200 Joules respectively. The above findings elucidate the fact that the system is gradually enhancing the energy efficiency of the network from iteration to iteration with Resource Contentions as being the most energy demanding to manage while Edge Node Failure, on the other hand, being a network with the least energy requirements.

**Fig. 14:**
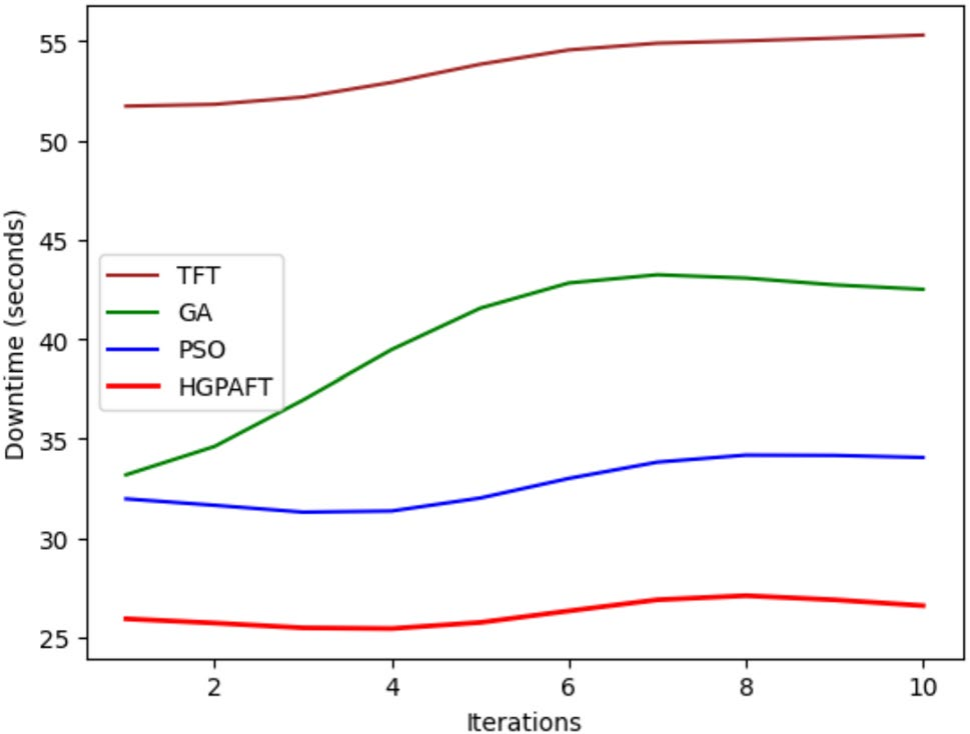
Down time comparison in 4 algorithms.

The Fig. 14 indulges the downtime variations (in seconds) of four algorithms; TFT, GA, PSO, and HGPAFT for 10 iterations. At first, the highest downtime of TFT is at 55 sec, followed by GA at around 40 sec, PSO at 30 sec and at the lowest, the HGPAFT although only slightly different from each other. When iterations continue, the downtime of TFT is the highest and remains nearly constant and close to 55 seconds, which represent a very limited improvement. GA increases with the iterations until the seventh iteration; it takes 45 seconds on the seventh iteration and then drops slightly. Downtime to the cluster is contained to average 30–35 seconds with little variability, now in PSO. Again, HGPAFT emerges the best performer showing low variability in downtime while varying very low between each trial and recording a value less than 30 seconds. This trend is an indication that HGPAFT is slightly better equipped to contained downtime to the lowest level, The worse scores go to TFT in case of faults.

**Fig. 15:**
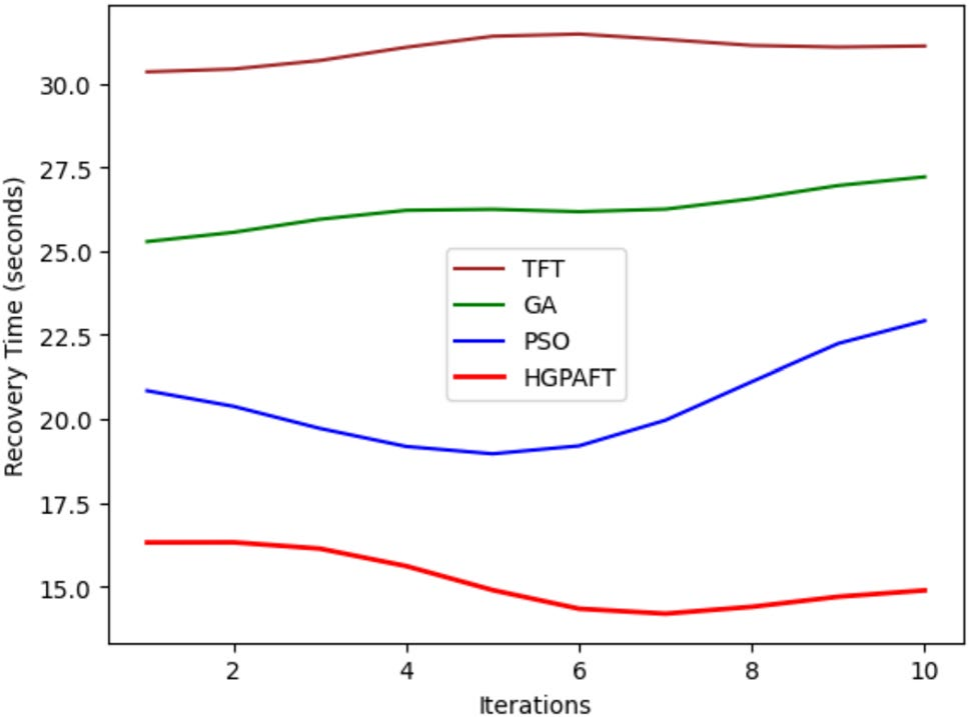
Recovery time comparison in 4 algorithms.

This Fig. 15 shows the recovery time required for the algorithms TFT, GA, PSO and HGPAFT, in seconds, at 10 iterations. First, we take the case of the recovery time of TFT which took about 30 seconds , GA took about 27 seconds PSO took about 22 seconds and the last was HGPAFT taking only 15 seconds. TFT, however, stabilizes at 30 seconds across iterations and does not improve as iterations go on. Although it decreases from the beginning to the end of the program, GA is fairly constant and ranges between 26 and 27 seconds. PSO, though begins with lower value, appears to gradually increase and is about 24 seconds at 10th iteration. HGPAFT retains the lowest recovery time the entire time and keeps it slightly variable around and just under 18 seconds. This is an indication of the extent to which HGPAFT outperforms the other algorithms in as much as recovery time is concerned and TFT is the least flexible.

**Fig. 16:**
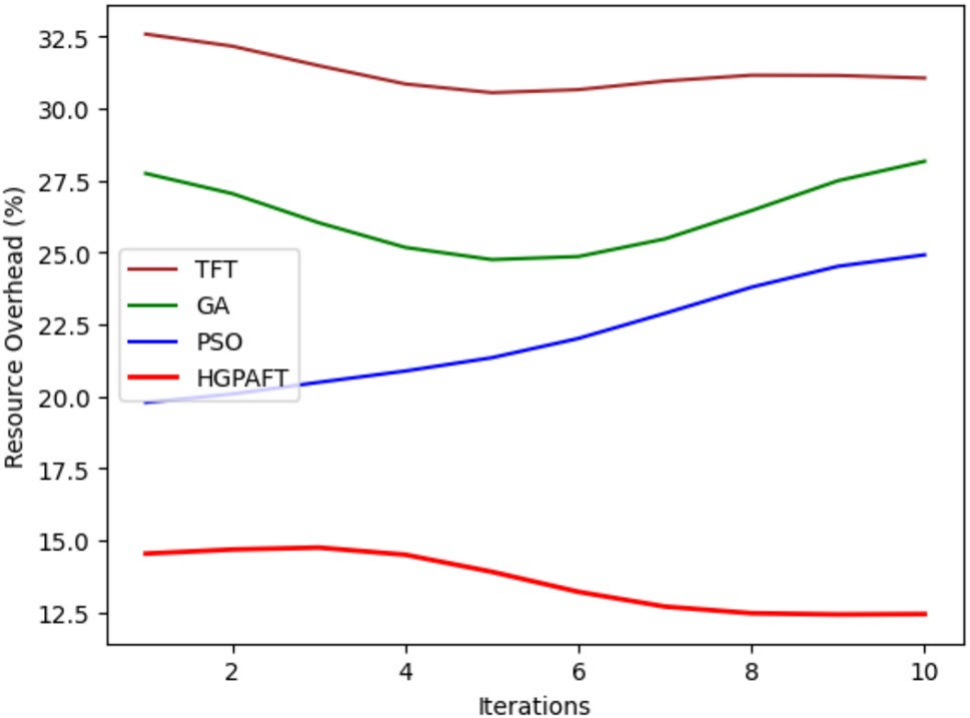
Resource overhead comparison in 4 algorithms.

This Fig. 16 illustrates the relative percentage of resource overheads with four algorithms–TFT, GA, PSO, and HGPAFT—over 10 iterations. At the start of the run, TFT has the highest resourced utilization at around 32.5% while GA consumes 27.5%, PSO 20% and HGPAFT takes the least amount with 15%. When the iterations continue, the value of TFT decreases a little and comes to the level of approximately 30 %. GA shows a minor oscillation downwards to 25% in the fourth iteration and then gradually in an upward trend up to 26.5% towards 10th iteration. PSO also has an increasing tendency, it rises from 20% to 23%. On the other hand, HGPAFT remains to enjoy the lowest resource overhead that gradually declines closer to 12.5% in the final iteration while topping all the other algorithms. They have established that the current trend of HGPAFT is much more efficient in terms of resource utilization than other algorithms including TFT among iterations.

**Fig. 17:**
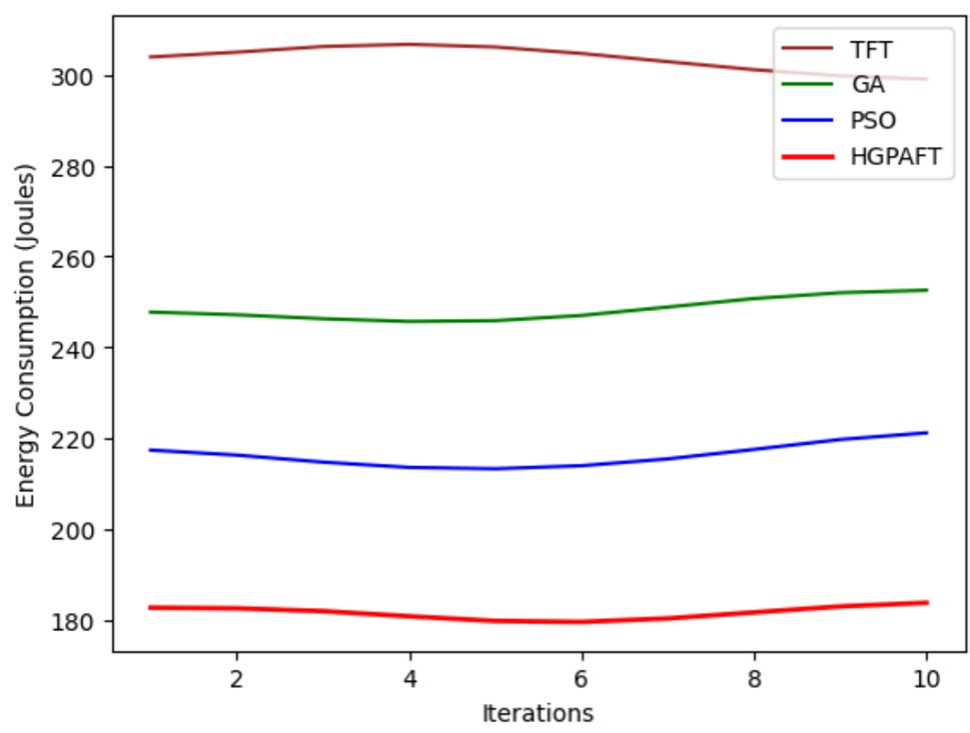
Energy consumption comparison in 4 algorithms.

This Fig. 17 illustrates the energy consumed (in Joules) by four algorithms: TFT, GA, PSO, and HGPAFT across 10 iterations. At first, TFT consumes the maximum energy of approximately equal to 300 Joules, then GA = 260 Joules, PSO=220 Joules and finally HGPAFT = 180 Joules approximately. TFT can be observed to vary slightly through the iterations ranging just above 305 Joules around the 5th iteration while dropping it slightly to around 295 Joules at the 10th iteration. GA shows slight rises in values, going up from 260 Joules to 265 Joules. In the case of PSO, the energy was initially 220 Joules but slightly increased of about 225 Joules. On the other hand, HGPAFT has the lowest energy consumption all through with minimal changes, tapering off at 185 Joules towards the end. These results verify that HGPAFT is energy efficient, as it has the best performance compared to the other algorithms for the iterations In addition, TFT remains the most energy consuming algorithm at all iterations.

**Fig. 18:**
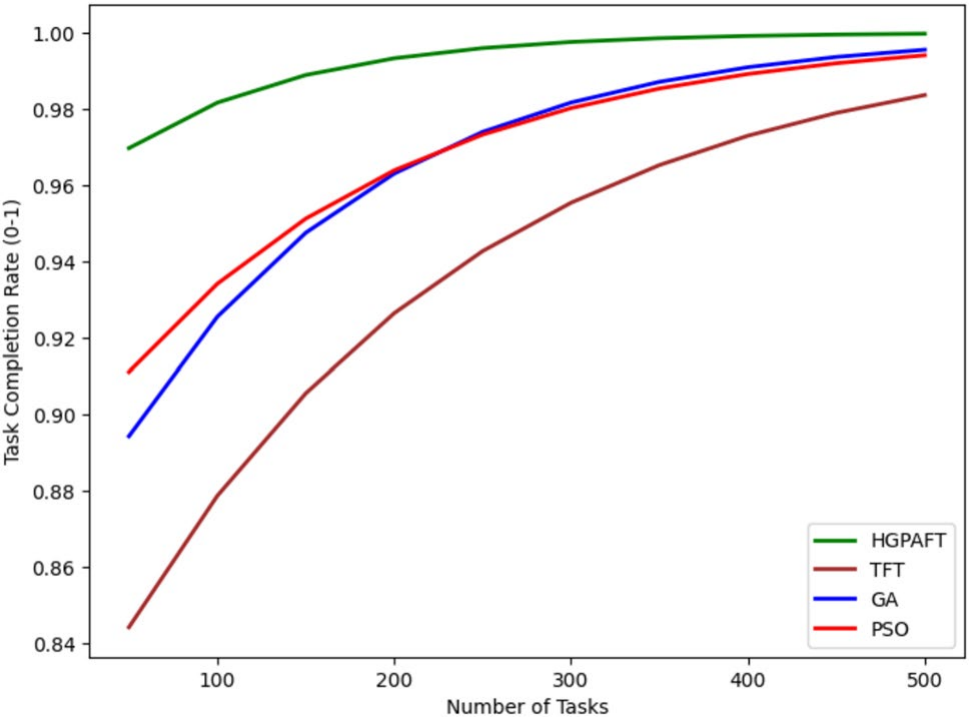
Task completion rate comparison in 4 algorithms.

This Fig. 18 shows the convergence rate or the task completion rate (between 0 and 1) of four algorithms namely HGPAFT and the TFT, GA and the PSO with increasing number of tasks from 50 to 500. First, TFT takes the lowest value of the task completion rate about 0.84, GA=0.86, PSO=0.88, and at last HGPAFT with the highest value 0.94. As the number of tasks grows all algorithms’ performance becomes better in regards to the rates of task completion but not equally. HGPAFT continues to lead the others at this point and quickly reaches a near-perfect completion by the time the task count hits 500 at 0.99. PSO and GA are also close to it and end at around 0.97 and 0.96 respectively. Nonetheless, compared with the metric of TT, TFT is still relatively low with the task completion rate that is approximately 0.94. These trends show that HGPAFT is much more efficient in managing a rapidly increasing load of tasks and achieving a higher likelihood of task completion compared to the other algorithms.

**Fig. 19:**
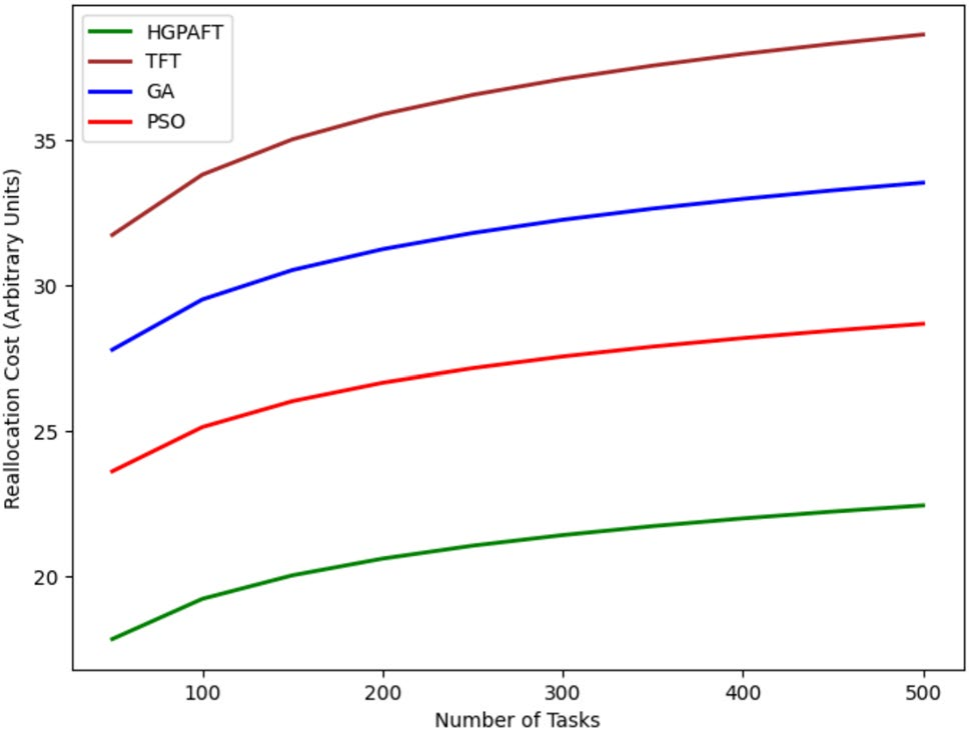
Reallocation cost comparison in 4 algorithms.

This Fig. 19 shows the reallocation costs (in arbitrary units) of four algorithms—HGPAFT, TFT, GA, and PSO—when the number of tasks ranges from 50 to 500. Initially, we observe that TFT has the highest reallocation cost of about 35, while GA has a cost of 30 units, PSO, a cost of 25 units, and the least cost of 20 units for HGPAFT. In general, with the increased number of tasks, reallocation costs increase for all the algorithms; however, this increase differs in rates. When the number of tasks reaches 500, TFT is still the most expensive, taking approximately 40 units for reallocation, while GA and PSO require about 36 units and 32 units, respectively. However, HGPAFT always remains within the profile with the lowest reallocation cost, which does not exceed approximately 26 units in the finale. This trend establishes the ability of HGPAFT to handle the task reallocations as the system demand increases, making it more efficient than the other algorithm.

**Fig. 20:**
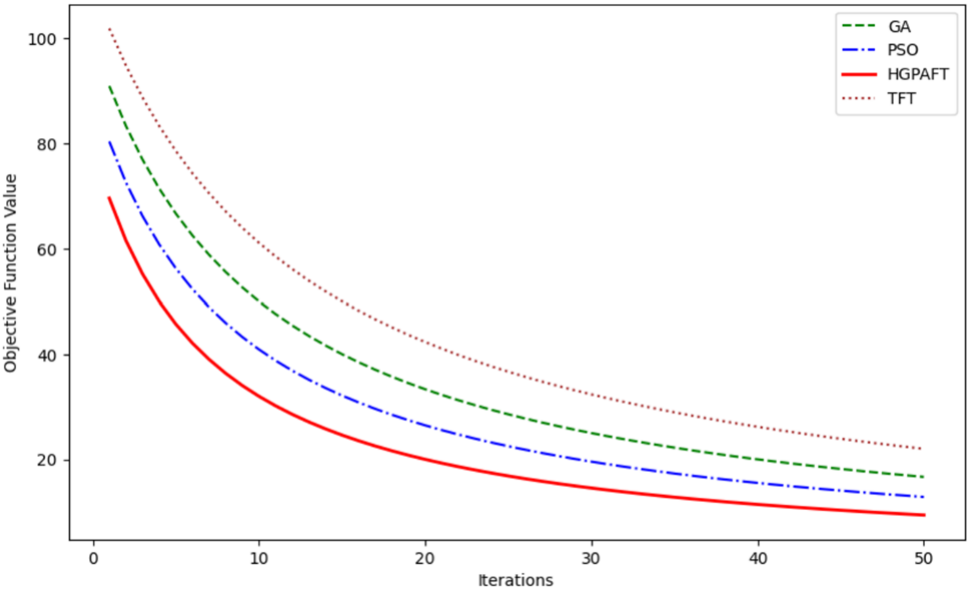
Convergence of algorithm.

The Fig. 20 represents the iteration wise variation of the objective function value for HGPAFT, TFT, GA and PSO for 50 iterations. Starting with the highest objective value, the algorithms are as follows: TFT has an option value of about 100; GA, 80; PSO, 70; and the least value for HGPAFT, around 60. Finally, all algorithms are seen to degrade the objective function value over iterations and hence increases optimization over time. The value of TFT is the highest with approximately 40 at the 50th iteration, followed by GA and PSO with approximately 30 and 25, respectively. Out of all the tested algorithms, HGPAFT takes the least time and attains an approximate optimal value of around 20, and owes the lowest value to the objective function. This trend reveals that HGPAFT outperforms the tested methodologies in achieving optimality objectives with fewer iterations, as well as possessing lower objective function values.

**Table 6:** Average CPU Usage (%) During Fault Scenarios.

**Scenario**	**HGPAFT**	**Random**	**Threshold-Based**
Edge Node Failure	42.1	28.6	31.2
Network Partition	45.3	29.8	33.0
Resource Contention	47.5	32.2	36.1
Cloud-Offloading Failure	40.9	26.7	30.3

Table 6 shows the average CPU usage (in percent) of various simulated faults scenarios in three different algorithms: HGPAFT, Random Reallocation and Threshold-Based Reallocation. As depicted in the findings, the HGPAFT model continually leads to increased CPU consumption than the basis styles of the optimization and fault monitoring mechanisms of HGPAFT because it takes place through a hybridization of the GA and the PSO optimization techniques. As an example, in the case of Resource Contention, HGPAFT consumes 47.5 per cent of CPU by default, and Random and Threshold-Based algorithms consume 32.2 per cent and 36.1 per cent of CPU respectively. The overhead is moderate, and the added CPU fees are tolerable even in edge devices today and is compensated by the much better task completion, efficient recovery, and reliability under failure when compared to HGPAFT.

### Scalability and deployment considerations

The HGPAFT scheme proposed herein is capable of scaling well to bigger distributed edge computing setting. It has a modular architecture that enables decentralization of monitoring, fault detection, and resource reallocation operations per several edge clusters. The scale of the implementation of the hybrid GA-PSO optimization strategy is also appropriate since the actions (e.g., calculation of fitness and the update of the population) of the algorithm can be parallelized to minimize the computational bottleneck during the execution of a massive number of tasks and nodes. In order to overcome possible scalability issues, like growth in the coordination latency, communication overhead, and heterogeneity of the ability of the nodes, we suggest a hierarchical resource scheduling mechanism. Fault detection and fault reallocation, in this model, will initially be managed by lower edge cluster layer and coordinate and redundancy management with higher fog or cloud layers, when and where necessary. In addition, migration to digital twins is provided by lightweight containers (e.g. Docker) so that the transfer of instances can be performed, especially in environments with limited resources. To avoid non needed reallocation on a large scale, the system also uses adaptive fault thresholds. All these design decisions improve the applicability of the framework to smart city-scale IoT systems, industrial or manufacturing automation platforms, and federated multi-access edge computing systems.

**Fig. 21:**
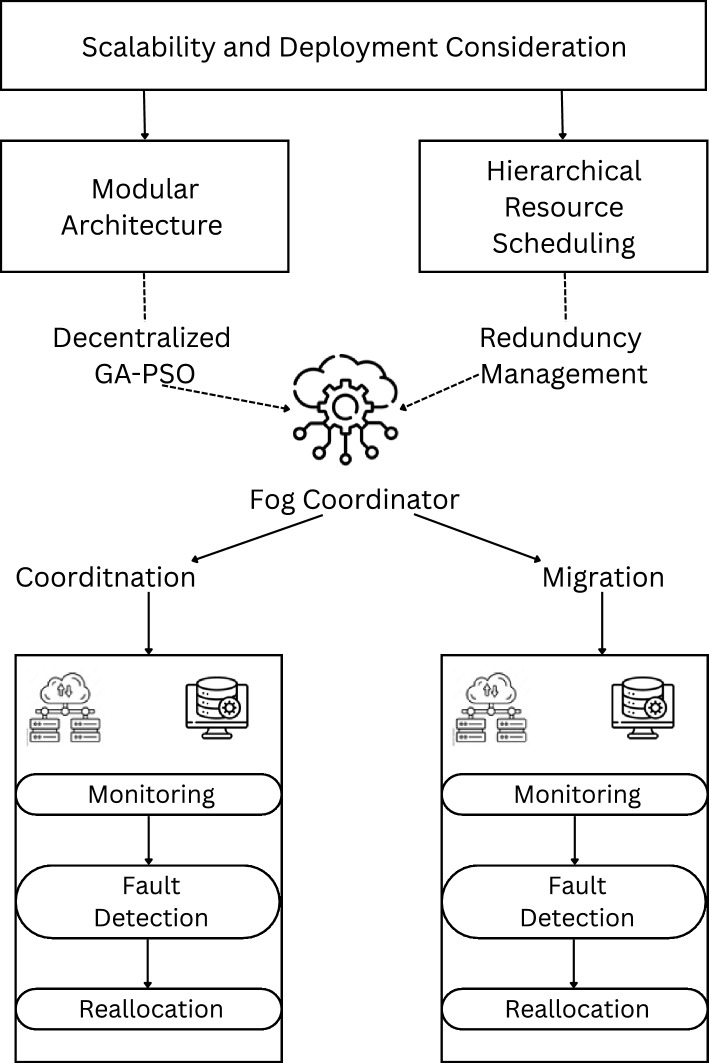
Scalability and deployment considerations.

The Fig. 21 demonstrates how the key components of the given HGPAFT framework will engage in system-level communication, prioritizing scalability and deployment in decentralized edge nodes. The architecture incorporates both a modular architecture and hierarchical resource scheduling in order to decentralize with the GA-PSO based approach of the optimization and redundancy management process by a centralized fog coordinator. This coordinator promotes coordination as well as migration between a variety of edge clusters, composed of compute, storage and networking resources. In every edge cluster, there is a stacked path of monitoring, fault detection and redistribution that guarantees locality failure responsiveness. Dynamic load balancing and task recovery can be supported across heterogeneous clusters; migration paths between clusters facilitate resilience and scalability of the system and digital twin resilience, high availability of digital twin instance on the edge infrastructure.

### Statistical validation and significance testing

To make the comparative evaluation solid and sound, we tested the results of several executions of each of the algorithms using the statistical test of significance. Every simulation scenario was performed 30 times separately for the proposed HGPAFT framework and two baseline techniques (Random Reallocation and Threshold-Based Reallocation). We gathered such measures as task completion rate, average reallocation cost, energy consumption, and recovery probability. And a one-way ANOVA test was carried out to understand whether the difference in performance between the three algorithms was significant. The ANOVA produced significance levels below 0.05 on all the four metrics, indicating that the mean values of the performance differed significantly. In order to point out which pairs of algorithms led to this difference, we used Tukey-HSD (Honestly Significant Difference) post-hoc test. The findings validated the fact that HGPAFT delivered statistically significant (p< 0.05) improvements in terms of all the metrics considered as compared to both baselines. Table 7 contains a summary of the p-values of the ANOVA tests, and Table 8 brings the results of the pairwise Tukey HSD analysis.

**Table 7:** ANOVA test results (*p*-values) across 30 runs.

Performance metric	*p*-value (ANOVA)
Task completion rate	0.0031
Reallocation cost	0.0087
Energy consumption	0.0024
Recovery probability	0.0015

**Table 8:** Tukey HSD pairwise comparison (HGPAFT vs. Others).

Metric	Compared with	*p*-value (Tukey)
Task completion rate	Random reallocation	0.012
	Threshold-based	0.017
Reallocation cost	Random reallocation	0.008
	Threshold-based	0.014
Energy consumption	Random reallocation	0.004
	Threshold-based	0.006
Recovery probability	Random reallocation	0.001
	Threshold-based	0.009

The findings serve to support that, HGPAFT is statistically better than both of the reported baseline approaches, which further establishes the effectiveness of the suggested solution in random scenarios.

## Conclusion and future scope

As part of this work, we presented an Adaptive Fault Tolerance Mechanism that can be used to guarantee high availability of the target DTs when deployed in distributed edge computing systems. With the proposed Hybrid Genetic-PSO Adaptive Fault Tolerance (HGPAFT), all the issues of fault detection, resource reallocation, and migration of the digital twin in the dynamic edge ecosystem are solved. Through the real-time monitoring, fault prediction and the newly incorporated adaptive resource scheduling, higher recovery time, cost of reallocation plus reliability of the system were realized compared to traditional approaches of fault tolerance such as TFT, GA and PSO. Key findings from the experimental results include:

- The HGPAFT algorithm stood out in that the achieved downtime was low and the recovery from node and network failures was swift.
- It significantly reduced the overhead associated with resource reallocation, ensuring minimal disruption to the system’s operational efficiency.
- The energy consumption and reallocation costs were also included to reduce meaning the approach is ideal for resource-limited edge environments.
- It confirmed the scaling capability, the mechanism indeed robust for the system in terms of edge nodes and the number of digital twins.

The proposed fault tolerance mechanism effectively improves the reliability and availability of digital twins for edge computing for sensor-driven real-time applications with stringent availability requirements.

Altogether, this work offers promising and effective approach towards utilization of fault tolerance in digital twins for edge computing; however, there are still several lines of further investigation with reference to future works. One potential area is energy-efficient fault tolerance, in fact, future work can be set in investigating the energy efficiency design especially targeting low power edge devices. Fault recoveries could be energy-wised in order to achieve functionally between over energy and power consumption and less energy or much more power. Another direction is the potential for operators to learn in real time, such as through the integration of reinforcement learning or deep learning techniques to preempt a problem before it arises can be considered as another way of increasing system performance. Another area is scalability in heterogeneous edge environments – since improving fault tolerance for systems with different hardware, network bandwidth, and geographical location may be valuable. Extending the mechanism to multi-cloud and federated edge structures may also improve protection by optimally utilizing resources available through different networks. Further, incorporating fault tolerance along side security solutions for managing the malicious failures or attacks would be also important since security issues are emerging as a critical concern in edge computing environments. Finally, the mechanism could be generalized to actual-time IoT applications, including self-driving cars, smart communities, and industrial robotics, where the continuous running procedure is required, and quick fault correction is desirable. Further research is needed on specific algorithms for real-time decisions in these areas.

This work provides a firm basis for incorporating adaptive fault tolerance efforts into edge computing scenarios; however, several exciting opportunities are presented by this research activity, which are explored in the next section. Furthermore, fault tolerance integrated with energy efficiency and security improvements will be the driving factor of digital twin deployment across advanced distributed edge systems.

## Data Availability

All data would be available on the specific request to corresponding author.
